# A Novel Dual URAT1/GLUT9 Inhibitor Reduces Hyperuricemia by Enhancing Uric Acid Excretion and Attenuating Renal Fibrosis

**DOI:** 10.3390/ph19030490

**Published:** 2026-03-16

**Authors:** Hailong Zhang, Jiaxin Huang, Wenji Yang, Wenhu Zhou, Jinsong Ding, Qianbin Li, Gaoyun Hu

**Affiliations:** 1 Xiangya School of Pharmaceutical Sciences, Central South University, Changsha 410013, China; 2 King-Eagle Med Technology Co., Ltd., Changsha 410221, China; 3 Hunan Provincial Transdermal Patch Drug Delivery System Engineering Technology Research Center, Changsha 410221, China

**Keywords:** hyperuricemia, HIPK2 inhibitor, uricosurics, URAT1, GLUT9, tubulointerstitial fibrosis

## Abstract

**Background**: Hyperuricemia (HUA) is a metabolic disorder that severely threatens human health. Chronic uric acid (UA) overload promotes the progression of tubulointerstitial fibrosis (TIF), leading to impaired UA excretion. Our previous studies identified HIPK2 inhibitor XRF-1021, which exhibits robust anti-TIF activity and lowers UA levels in vivo. This study aimed to elucidate its UA-lowering mechanism and therapeutic potential for HUA. **Methods**: Uricase and xanthine oxidase (XOD) assays were performed to assess effects on UA degradation/production. HEK293T cells transiently expressing UA transporters and gene-knockdown rats were used to evaluate transporter inhibition, while HK-2 cells were analyzed by Western blot. Pharmacokinetics were characterized in rats. Efficacy was tested in potassium oxonate-induced acute HUA rats, diet/adenine-induced chronic HUA quails, and adenine-induced mice with HUA secondary to TIF. Maximum tolerated dose and long-term toxicity were assessed in rats. **Results**: XRF-1021 neither activated uricase nor inhibited XOD, indicating no direct effect on UA catabolism or synthesis. Instead, XRF-1021 inhibited URAT1 and GLUT9, reducing renal UA reabsorption, while sparing OAT3, OAT4, and ABCG2 activity and upregulating OAT3 and NPT4, suggesting minimal risk of disrupting drug or uremic toxin handling. XRF-1021 showed dose-dependent systemic exposure in rats, lowered serum UA, and provided renal protection in vivo. LD_50_ values were 2345.4 mg/kg (male) and 1078.9 mg/kg (female), with no obvious toxicity after long-term dosing. **Conclusions**: XRF-1021 lowers UA by inhibiting URAT1 and GLUT9 to enhance renal UA excretion and provides kidney protection, supporting XRF-1021 as a promising candidate for HUA therapy.

## 1. Introduction

Hyperuricemia (HUA) is a metabolic disorder characterized by elevated serum uric acid (UA) levels due to abnormal purine metabolism or impaired UA excretion [1], and it threatens multiple organs including joints [2], kidneys [3], and the cardiovascular system [4]. Its principal pathological basis is chronic UA overload, which drives monosodium urate (MSU) crystal deposition and local inflammation, progressively damaging tissues and impairing function [5]. Importantly, the kidney, as the primary organ responsible for UA excretion, is particularly vulnerable to persistent hyperuricemic stimulation [6]. Excessive UA exposure damages proximal tubular epithelial cells, which in turn activate autocrine and paracrine signaling pathways that promote the progression of tubulointerstitial fibrosis (TIF), thereby exacerbating renal dysfunction [7,8]. Therefore, restoring the balance between UA production and excretion, lowering serum UA, and protecting renal function are key therapeutic strategies.

Pharmacological therapy remains the mainstay for HUA. Clinically used small-molecule UA-lowering agents are mainly classified into UA synthesis inhibitors and uricosurics [9,10]. Febuxostat (FEB) and allopurinol are classical xanthine oxidase inhibitors (XOIs) that effectively reduce UA overproduction with long-term efficacy [11]. However, the high prevalence of the HLA-B*58:01 allele in East and Southeast Asian populations (reported at 10.6~16.4% in multiple cohorts) is strongly associated with allopurinol-induced severe hypersensitivity reactions [12], which substantially limits allopurinol use in these populations. In addition, owing to its pharmacological profile and cardiovascular safety concerns, FEB has been associated with an increased risk of cardiovascular and all-cause mortality in gout patients with established cardiovascular disease [13,14], leading the U.S. Food and Drug Administration (U.S. FDA) to issue a boxed warning. Collectively, these limitations have created a clinical bottleneck for XOIs and underscore the need for alternative UA-lowering strategies.

For patients with decreased UA excretion who cannot tolerate XOIs, uricosurics represent an important therapeutic alternative and are increasingly becoming a focus of innovative drug development. These agents act by inhibiting renal tubular UA transporters, primarily urate transporter 1 (URAT1) and glucose transporter 9 (GLUT9), thereby reducing UA reabsorption and lowering serum UA levels [15]. However, uricosurics carry safety concerns due to off-target effects. For example, benzbromarone (BEM), a potent URAT1 inhibitor, also blocks organic anion transporter 1 (OAT1) and organic anion transporter 3 (OAT3), potentially causing drug accumulation and worsening renal dysfunction [16], and has also been associated with severe hepatotoxicity [17]. Another URAT1 inhibitor, lesinurad (LES), often requires XOIs co-administration [18], but this combination increases renal risk [19]. Moreover, uricosuric efficacy depends on preserved renal function, yet they lack direct renoprotective effects. Thus, optimizing molecular design to improve target selectivity while promoting renal recovery is critical for advancing HUA therapy.

**Figure 1:**
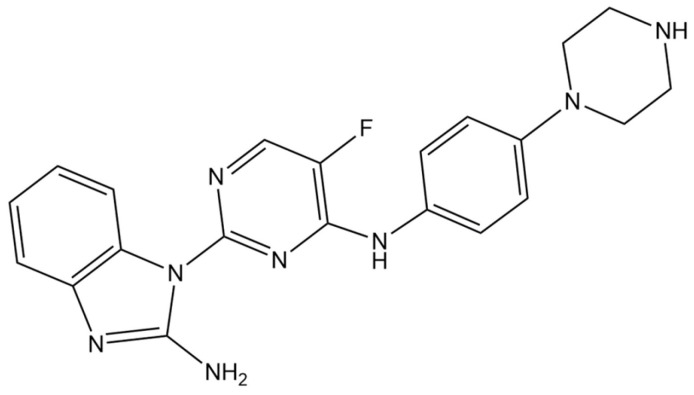
The chemical structure of XRF-1021.

Notably, clinical studies have revealed a strong link between TIF and HUA [20]. UA and xanthine oxidase (XOD)-mediated production of reactive oxygen species (ROS) injure renal endothelial cells, causing local ischemia, interstitial damage, and renal dysfunction [21]. In addition, elevated UA also promotes excessive ROS production in tubular epithelial cells, activating inflammatory pathways, cytokine release, and immune cell infiltration, thereby accelerating fibrosis [22]. Recent evidence highlights homeodomain-interacting protein kinase 2 (HIPK2) as an amplifier of TGF-β/Smad3 signaling, which activates Wnt/β-catenin, induces epithelial–mesenchymal transition (EMT), and upregulates inflammatory mediators, reinforcing a profibrotic microenvironment [23,24]. In our previous work, we identified HIPK2 as a therapeutic target and, guided by the core scaffold of the prototypical inhibitor TAE-226 [25], designed and synthesized a series of HIPK2 inhibitors. Among these candidates, a 2,4-disubstituted 5-fluoropyrimidine derivative named XRF-1021 (Figure 1) demonstrated pronounced UA-lowering activity in an adenine-induced renal fibrosis mouse model [26]. Building on these findings, we conducted a series of studies to evaluate the UA-lowering efficacy and renoprotective properties of XRF-1021.

## 2. Results

### 2.1. XRF-1021 Is Neither a Uricase Activator nor an XOD Inhibitor

**Figure 2:**
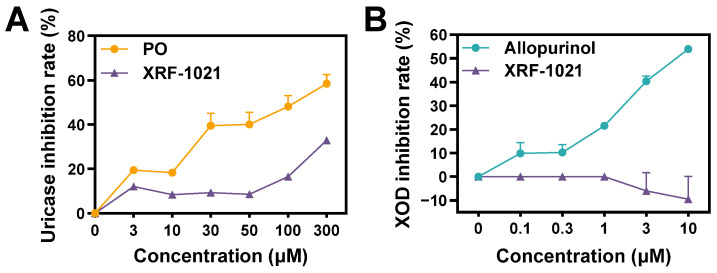
(**A**) Inhibition of uricase activity by PO or XRF-1021 at different concentrations (*n* = 3). (**B**) Inhibition of XOD activity by allopurinol or XRF-1021 at different concentrations (*n* = 3). Data are presented as mean ± SD.

First, we evaluated whether XRF-1021 modulates uricase activity. Although humans lack functional uricase, rodents express uricase, which oxidizes UA to allantoin and thereby lowers circulating UA levels [27]. To exclude the possibility that the UA-lowering effect of XRF-1021 in rodent models was attributable to activation of endogenous uricase, we assessed its impact on uricase activity. As shown in Figure 2A, unlike potassium oxonate (PO), XRF-1021 produced only minimal inhibition of uricase across 3–100 μM, with inhibition remaining around 15%. At 300 μM, uricase inhibition increased to 56.3% of that observed with PO, suggesting that XRF-1021 is a weak uricase inhibitor rather than an activator. These results further indicate that UA reduction in rodents was not driven by enhancement of endogenous uricase activity.

Next, we examined the inhibitory effect of XRF-1021 on XOD, the key enzyme catalyzing the final steps of purine catabolism to UA, to determine whether XRF-1021 reduces UA generation at the source. As expected, allopurinol inhibited XOD in a concentration-dependent manner, achieving >50% inhibition at 10 μM (Figure 2B). In contrast, XRF-1021 showed no detectable inhibitory activity against XOD under the same conditions. Together, these findings demonstrate that XRF-1021 does not act as a uricase activator or an XOIs, supporting an alternative mechanism—most plausibly, the facilitation of renal UA elimination via the inhibition of UA transporters.

### 2.2. Inhibition of URAT1/GLUT9 by XRF-1021 to Block UA Reabsorption and Transport into Bloodstream

To determine whether XRF-1021 enhances renal UA elimination by inhibiting UA reabsorption via UA transporters, we first examined its effect on the activity of URAT1, the principal transporter mediating UA reabsorption.

**Figure 3:**
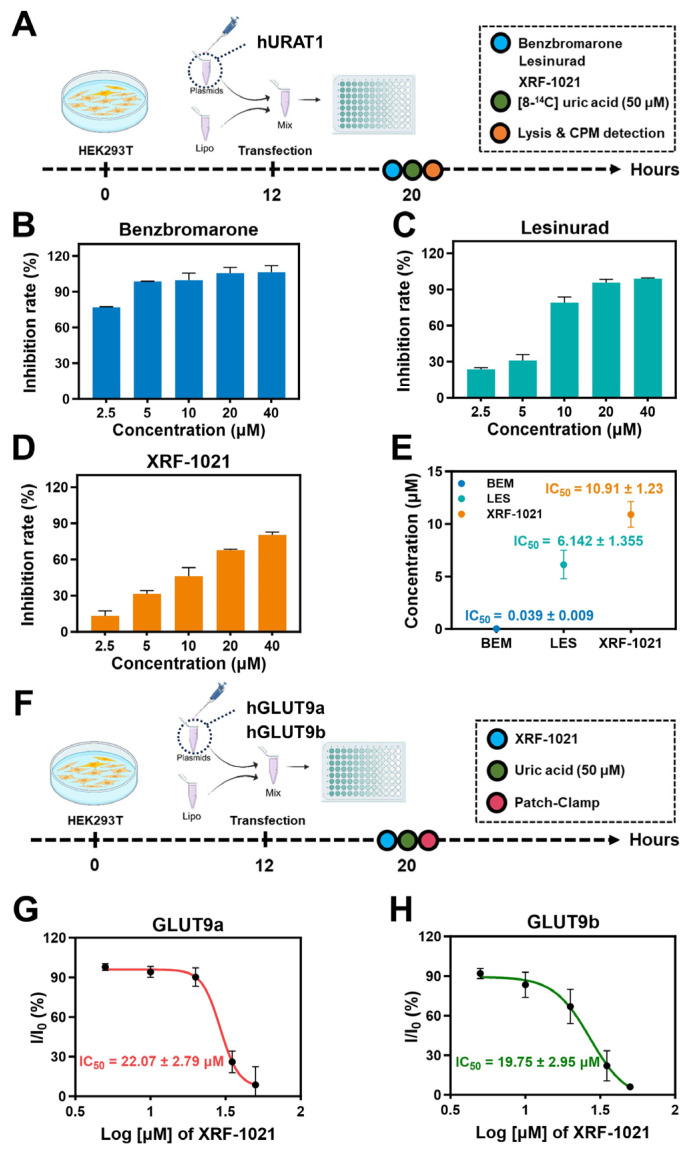
(**A**) Schematic representation of the experimental protocol of [8-^14^C] UA uptake assays. Inhibition rates of URAT1 by (**B**) BEM, (**C**) LES, and (**D**) XRF-1021, at varying concentrations in HEK293T cells. (**E**) URAT1 IC_50_ value of XRF-1021, BEM and LES in HEK293T cells (*n* = 2). (**F**) Schematic representation of the experimental protocol of patch-clamp experiments. The inhibitory rates kinetics of (**G**) GLUT9a and (**H**) GLUT9b (*n* = 3). Data are presented as mean ± SD.

URAT1 is a key transporter located on the apical membrane of renal proximal tubules, plays a critical role in maintaining systemic UA homeostasis [28]. The transient transfected hURAT1-expressing HEK293 cell line was established, and the cells were pretreated with increasing concentrations of BEM, LES, or XRF-1021, followed by co-incubation with ^14^C-labeled UA. Intracellular UA uptake was quantified, and inhibition rates were calculated (Figure 3A). The results demonstrated that all three compounds suppressed UA transport in a clear dose-dependent manner (Figure 3B–D). As classical URAT1 inhibitors, BEM and LES inhibited URAT1-mediated UA uptake with IC_50_ values of 0.039 ± 0.009 μM and 6.142 ± 1.355 μM, respectively, consistent with their reported pharmacology. XRF-1021 also inhibited UA uptake, with an IC_50_ of 10.91 ± 1.23 μM. Accordingly, the URAT1 IC_50_ of XRF-1021 was ~280-fold higher than that of BEM and ~1.8-fold higher than that of LES, indicating URAT1 inhibition comparable to LES but substantially weaker than BEM (Figure 3E), this solution indirectly suggests that XRF-1021 may possess multi-target inhibitory activity, confirming its functional activity against URAT1 in vitro.

GLUT9, a member of the major facilitator superfamily (MFS), is expressed on both the basolateral and apical membranes of renal proximal tubules, where it mediates UA efflux from the cytoplasm to the bloodstream. Given the critical role of GLUT9 in renal UA handling, we further examined the effects of XRF-1021 on both isoforms of this transporter. GLUT9a, expressed on the basolateral membrane, facilitates UA efflux from tubular cells to the renal interstitium and circulation, whereas GLUT9b, predominantly localized to the apical membrane, mediates UA uptake from the tubular lumen [29,30]. Transient transfected hGLUT9a- and hGLUT9b-expressing HEK293 cell lines were generated to assess isoform-specific responses (Figure 3F). XRF-1021 inhibited UA transport mediated by both isoforms, with IC_50_ values of 22.07 ± 2.79 μM for GLUT9a and 19.75 ± 2.95 μM for GLUT9b (Figure 3G–H), indicating that XRF-1021 exhibits potent inhibitory effects on both GLUT9 subtypes.

**Figure 4:**
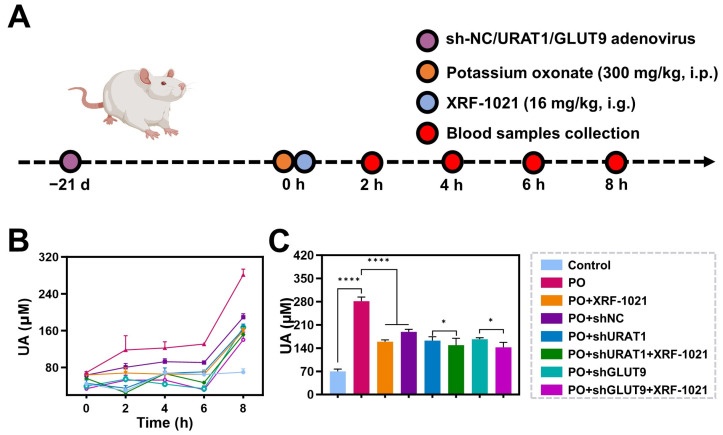
(**A**) Schematic representation of the experimental protocol of knocked down URAT1 or GLUT9 expression in rat kidney tissue. (**B**) The kinetic curve of blood UA level in rats with different treatments (*n* = 4). (**C**) The UA level of rats with different treatments at 8 h (*n* = 4). Data are presented as mean ± SD. Normality and homogeneity of variances were satisfied. Statistical significance was determined by one-way ANOVA followed by Dunnett’s *t*-test (* *p* < 0.05, **** *p* < 0.0001).

Furthermore, we knocked down URAT1 or GLUT9 expression in rat kidney tissue using shRNA-expressing adenovirus and elevated systemic UA levels via intraperitoneal PO (Figure 4A). By monitoring serum UA dynamics within 8 h after oral dosing, we further evaluated the dual-target inhibitory activity of XRF-1021 (Figure 4B). In PO-treated rats, serum UA increased progressively and reached ~4-fold of the control level at 8 h. In contrast, URAT1 or GLUT9 knockdown decreased serum UA by 42.1% and 40.7%, respectively, suggesting that these transporters exert comparable effects on UA handling in this model. Importantly, XRF-1021 administration further reduced serum UA by 7.06% and 16.17% in URAT1- and GLUT9-knockdown hyperuricemic rats, respectively, compared with the corresponding knockdown-only groups (Figure 4C). Collectively, these findings indicate that XRF-1021 inhibits both URAT1 and GLUT9 in vivo, supporting its classification as a dual URAT1/GLUT9 inhibitor.

### 2.3. Molecular Docking and Molecular Dynamics Simulations of XRF-1021 Against URAT1 and GLUT9

Uricosuric agents (e.g., BEM and LES) can occupy the UA-binding pocket of UA transporters through multiple hydrophobic interactions, thereby preventing the conformational changes required for UA transport. We therefore performed in silico molecular docking to characterize the binding poses of XRF-1021 within URAT1 and GLUT9 and to delineate the major hydrophobic interaction patterns at their putative binding sites.

**Figure 5:**
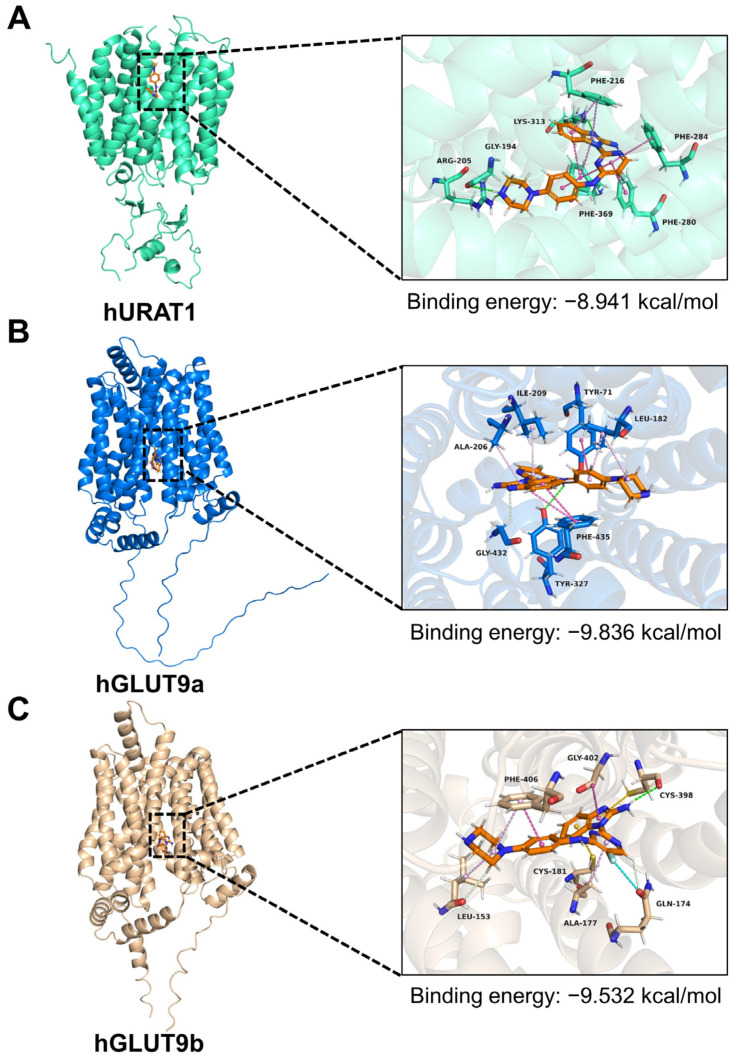
Molecular docking results of XRF-1021. Docking results of XRF-1021 with (**A**) hURAT1 (PDB ID: 9JDY), (**B**) hGLUT9a and (**C**) hGLUT9b in AutoDock Vina 1.2.5. Orange lines represent ligand molecules. Green lines, blue lines and gold lines represent amino acid residues.

**Figure 6:**
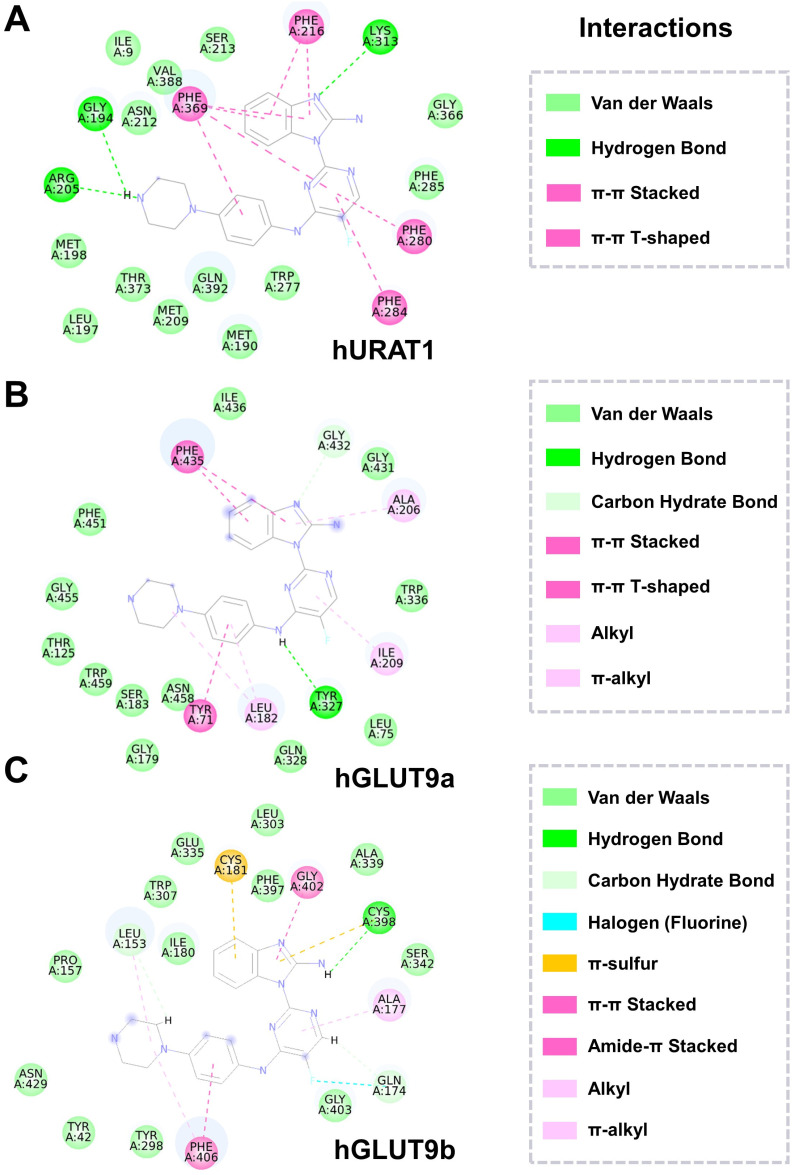
Overall binding interactions of XRF-1021 with (**A**) hURAT1, (**B**) hGLUT9a, and (**C**) hGLUT9b.

As an organic anion transporter, URAT1 facilitates UA reabsorption by alternating between outward-open binding and inward-open release conformations [31]. Molecular docking analysis revealed that XRF-1021 inserts into the central channel formed between the transmembrane domains of URAT1, with a binding energy of −8.914 kcal/mol, suggesting stable occupancy of the canonical UA-binding site (Figure 5A). Within this channel, XRF-1021 establishes multiple stabilizing interactions (Figure 6A): hydrogen bonds with Gly194, Lys313, and Arg205 residues, π–π stacking interactions with Phe396 and Phe216, and π–π T-shaped interactions with Phe369, Phe280, and Phe284. These residues are directly involved in UA recognition and transport, indicating that XRF-1021 can inhibit UA transport by occupying the canonical binding pocket of URAT1, forming a stable complex that blocks essential conformational changes.

The GLUT9 comprises 12 transmembrane helices and functions through a “rocker–switch” mechanism, alternating between outward- and inward-open conformations to enable UA translocation [32]. Using AlphaFold3 structural predictions, we modeled GLUT9a and GLUT9b and performed molecular docking with XRF-1021 [33,34]. The results showed that XRF-1021 was deeply embedded in the central pocket of GLUT9a with a binding energy of –9.836 kcal/mol (Figure 5B). The piperazine –NH– of XRF-1021 formed a hydrogen bond with Tyr327, anchoring the compound at the pocket entrance. In addition, XRF-1021 engaged in multiple stabilizing interactions: a carbon–hydrogen bond with Gly432, π–π stacking with Phe435, and a π–π T-shaped interaction with Tyr71. Furthermore, hydrophobic residues including Leu182, Ala206, and Ile209 contributed strong alkyl/π–alkyl contacts, providing a hydrophobic lock that reinforced ligand binding (Figure 6B). Together, these interactions indicate that XRF-1021 stabilizes GLUT9 in a locked conformation, thereby preventing the conformational transitions required for UA transport.

Similarly, XRF-1021 was found to bind deeply within the central pocket of GLUT9b with a binding energy of –9.532 kcal/mol, comparable to its interaction with GLUT9a (Figure 5C). The piperazine –NH– of XRF-1021 formed a hydrogen bond with Cys398 at the pocket base, while additional carbon–hydrogen (Leu153, Gln174) and halogen (Gln174) interactions further stabilized the complex. Moreover, Phe406 and Gly402 contributed strong aromatic “locks” through π–π and amide–π stacking, respectively, whereas hydrophobic residues such as Leu153, Ala177, and Phe406 reinforced binding via alkyl/π–alkyl interactions, creating a stable hydrophobic platform (Figure 6C). These interactions indicate that XRF-1021 firmly occupies the canonical UA-binding pocket of GLUT9b, thereby inhibiting its activity and reducing UA reabsorption.

To further validate the binding poses of the three complexes predicted by AutoDock Vina v1.2.5 and to evaluate their dynamic stability under physiological solution conditions, we performed 50-ns molecular dynamics simulations in GROMACS starting from the top-ranked docking conformations. The RMSD trajectories of the URAT1–, GLUT9a–, and GLUT9b–XRF-1021 complexes rapidly reached a plateau after ~10 ns, with mean RMSD values stabilizing at approximately 5.0, 2.5, and 3.0 Å, respectively, indicating system equilibration and a stable ligand conformation within the binding pocket (Figure S1A–C). Consistently, RMSF analysis showed that most residues in all three proteins fluctuated around ~2.0 Å, further supporting overall structural stability during the simulations (Figure S1D–F). Hydrogen bonding is a key noncovalent interaction contributing to protein–ligand complex stability. During the production phase, the average numbers of hydrogen bonds between XRF-1021 and URAT1, GLUT9a, and GLUT9b were 1.364, 1.026, and 1.753, respectively, suggesting an important role for polar interactions (Figure S1G–I). Notably, the average number of hydrogen bonds in the URAT1– and GLUT9b–XRF-1021 complexes was higher than that in the GLUT9a complex. This pattern implies that XRF-1021 binding to GLUT9a relies more heavily on hydrophobic contacts and van der Waals interactions, consistent with the docking results.

The above results collectively demonstrate that XRF-1021 can exert its pharmacological activity through stable binding to URAT1, GLUT9a, and GLUT9b via multiple interactions.

### 2.4. XRF-1021 Exhibits No Significant Inhibitory Activity on OAT3, OAT4 and ABCG2 but Upregulates the Expression of OAT3, OAT4 and NPT4

We next evaluated whether XRF-1021 influenced other major UA transporters beyond URAT1 and GLUT9. OAT3, organic anion transporter 4 (OAT4), and ATP-binding cassette, sub-family G, member 2 (ABCG2), widely expressed in the kidney and intestine, also contribute to UA and toxin excretion [35]. However, non-selective inhibition of these transporters may cause safety issues rather than therapeutic benefits. For example, BEM potently inhibits OAT3, impairing the clearance of nephrotoxins such as indoxyl sulfate and accelerating TIF. Similar concerns exist for ABCG2 [36] and OAT4 [37,38] inhibition, underscoring the need for selective targeting.

**Figure 7:**
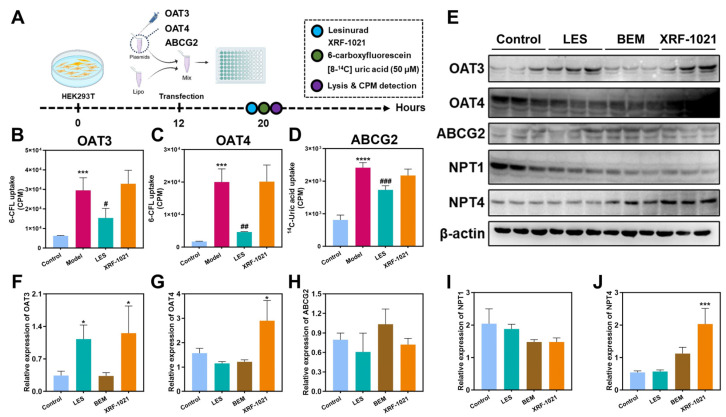
(**A**) Schematic representation of the experimental protocol of 6-CFL or [8-^14^C] UA uptake assays. Cellular 6-CFL levels in (**B**) OAT3-, (**C**) OAT4-, and [8-^14^C] UA levels in (**D**) ABCG2-transfected cells upon exposure to LES or XRF-1021, each at 10 µM, for 20-min exposure durations (*n* = 3). (**E**) Western blot analysis of transporter protein expression in HK-2 cells with different treatments (LES, BEM, and XRF-1021), and corresponding quantification of (**F**) OAT3, (**G**) OAT4, (**H**) ABCG2, (**I**) NPT1, and (**J**) NPT4 (*n* = 3). Data are presented as mean ± SD. Normality and homogeneity of variances were satisfied. Statistical significance was determined by one-way ANOVA followed by Dunnett’s *t*-test (* *p* < 0.05, *** *p* < 0.001, **** *p* < 0.0001, vs. Control; ^#^ *p* < 0.05, ^##^ *p* < 0.01, ^###^ *p* < 0.001 vs. Model).

To evaluate the target selectivity of XRF-1021, we established transient transfected HEK293T cell lines expressing OAT3, OAT4, or ABCG2 and systematically assessed the inhibitory effects of the compound (Figure 7A). The results demonstrated that XRF-1021 exhibited no significant inhibitory activity against these transporters within its therapeutic concentration range (Figure 7B–D), indicating a favorable selectivity profile. More importantly, Western blot analysis was performed to detect the transporters localized to the apical membrane of renal tubules that contributes to UA secretion, including OAT3, OAT4, ABCG2, NPT1, and NPT4 (Figure 7E), the uncropped original blot images are shown in Figure S2. The results showed that XRF-1021 markedly upregulated the protein expression of OAT3, OAT4, and NPT4, whereas no significant changes were observed in the protein levels of ABCG2 or NPT1 (Figure 7F–J). This suggests that XRF-1021 not only avoids interference with non-target transporters but may also synergistically enhance renal excretion pathways for both UA and uremic toxins. These findings highlight that XRF-1021 selectively inhibits URAT1 and GLUT9 without compromising the function of other key transporters, while potentially augmenting renal excretion mechanisms. Such a profile confers favorable safety characteristics and supports the clinical translational potential of XRF-1021.

### 2.5. Preliminary Evaluation of the Absorption and Tissue Distribution of XRF-1021 in Sprague–Dawley Rats

**Figure 8:**
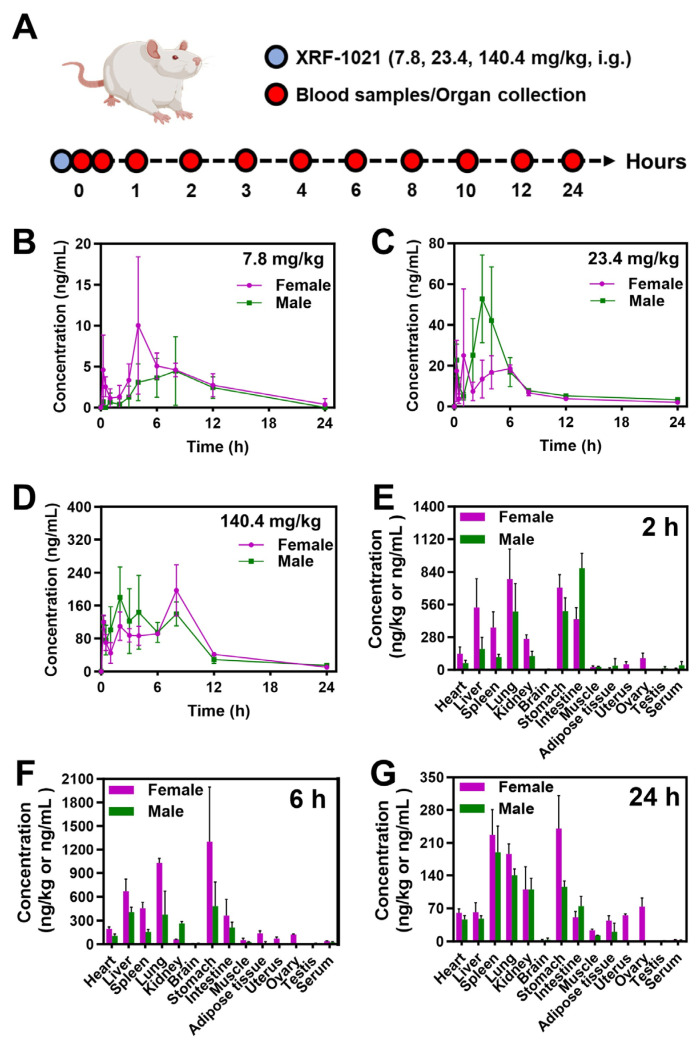
(**A**) Schematic of treatment regimen for pharmacokinetic research. Plasma concentration–time curves of XRF-1021 in SD rats after oral administration at doses of (**B**) 7.8, (**C**) 23.4, and (**D**) 140.4 mg/kg (*n* = 3). Tissue distribution of XRF-1021 in SD rats at (**E**) 2 h, (**F**) 6 h, and (**G**) 24 h following a single oral dose of 23.4 mg/kg (*n* = 3). Data are presented as mean ± SD.

Before evaluating the in vivo pharmacodynamic efficacy of XRF-1021 in HUA, we performed a preliminary pharmacokinetic assessment in Sprague–Dawley (SD) rats. XRF-1021 was orally administered to SD rats at 7.8, 23.4, and 140.4 mg/kg, and plasma samples were collected at predefined time points to characterize its absorption profile (Figure 8A). The single-dose pharmacokinetic parameters are summarized in Table 1. Following oral gavage of XRF-1021 at the three dose levels (dose ratio: 1:3:18), the corresponding dose-normalized fold changes in peak plasma concentration (C_max_) were 1.0:10.4:31.8, and those in AUC_0–t_ were 1.0:7.4:27.0. These results indicate that systemic exposure to XRF-1021 increased with escalating dose in SD rats, with no apparent sex-related differences (Figure 8B–D).

Following a single 23.4 mg/kg dose, tissue distribution was quantified at 2, 6, and 24 h (Figure 8E–G). At 2 and 6 h, XRF-1021 was most abundant in the stomach, followed by the small intestine, which is consistent with the observed T_max_ in both male and female SD rats and suggests ongoing gastrointestinal absorption during this window. At later time points (10 and 24 h), XRF-1021 was primarily distributed via the circulation to the spleen, lung, kidney, and liver, indicating relatively prolonged retention in the lung and kidney. This distribution profile may be advantageous for interventions targeting pulmonary and renal diseases. In contrast, XRF-1021 levels were lowest in the brain and testes, suggesting limited penetration across the blood–brain barrier and the blood–testis barrier. Notably, comparatively high concentrations were detected in the uterus and ovary, warranting further evaluation of potential reproductive toxicity in subsequent studies.

### 2.6. XRF-1021 Effectively Reduces Serum UA Levels in PO-Induced Acute HUA Rats

Acute HUA is characterized by the rapid elevation of serum UA due to complex clinical factors, often leading to kidney injury, electrolyte imbalance, and acid–base disturbances [39]. PO, a uricase inhibitor, induces a transient increase in UA after a single intraperitoneal injection in rats, followed by a gradual decline over time. This model therefore enables rapid assessment of the UA-lowering efficacy of XRF-1021.

**Figure 9:**
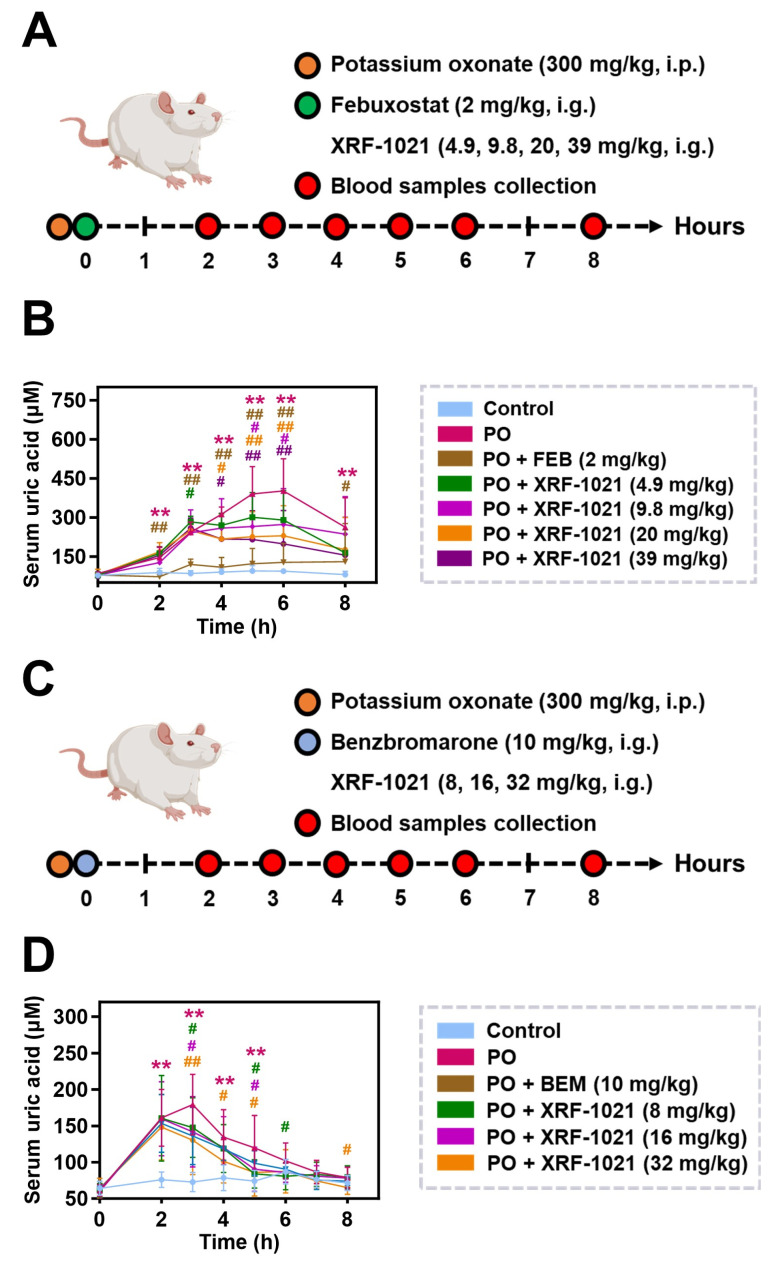
(**A**,**C**) Schematic of treatment regimen for acute HUA rats model. (**B**,**D**) The kinetic curve of blood UA level (*n* = 8). Data are presented as mean ± SD. Normality and homogeneity of variances were satisfied. Statistical significance was determined by one-way ANOVA followed by Dunnett’s *t*-test (** *p* < 0.01, vs. Control; ^#^ *p* < 0.05, ^##^ *p* < 0.01, vs. PO).

We first used the clinically established XOIs FEB (2 mg/kg) as a positive control to compare the UA-lowering potency of XRF-1021 and to define its minimal effective dose in PO-induced HUA rats (Figure 9A). Relative to normal rats, PO-treated animals exhibited a marked elevation in serum UA at 2 h post-induction, which remained substantially increased up to 6 h, confirming successful model establishment. FEB produced the rapid onset of action, significantly decreasing serum UA as early as 2 h after dosing, with >50% UA reduction maintained throughout 2–8 h. This rapid and robust effect is consistent with its mechanism of directly blocking UA production via XOIs. In contrast, XRF-1021 (4.9–39 mg/kg) showed a delayed onset, with significant UA reduction emerging at 4 h post-dose. At 6 h, XRF-1021 at 9.8, 20, and 39 mg/kg reduced serum UA by 32.0%, 42.9%, and 50.7%, respectively, relative to the PO group, thereby narrowing the efficacy gap versus FEB. The difference in onset is attributable to distinct mechanisms: FEB suppresses UA formation upstream, whereas XRF-1021 acts as a uricosuric agent that promotes UA excretion after UA has been generated, resulting in a lagged pharmacodynamic response. Based on these data, the minimal effective dose of XRF-1021 in this acute HUA model was 4.9 mg/kg, achieving a 15–50% reduction in serum UA between 4 and 8 h post-dose. Moreover, an XRF-1021 dose of 9.8 mg/kg was estimated to provide an approximately comparable UA-lowering effect to FEB under these conditions, although FEB remained more potent overall (Figure 9B).

We next compared XRF-1021 with BEM in the same model (Figure 9C). Compared with the control group, PO-treated animals showed a marked increase in serum UA at 3 h post-induction, followed by a gradual decline. This pattern is likely attributable to inter-individual variability in uricase activity and the relatively short effective exposure time of PO in vivo, which limits sustained uricase inhibition and thus prevents long-term maintenance of high serum UA levels [40]. XRF-1021 and BEM exhibited broadly similar efficacy with respect to the onset, duration, and magnitude of UA lowering. Both agents began to reduce UA at ~3 h. At that time point, 10 mg/kg BEM decreased serum UA by 24.1%, whereas XRF-1021 at 8, 16, and 32 mg/kg produced reductions of 17.6%, 20.8%, and 27.3%, respectively. Importantly, from 4 to 6 h post-dose, both 10 mg/kg BEM and 8–32 mg/kg XRF-1021 maintained significant UA-lowering effects (approximately 10–30%) with comparable duration (Figure 9D).

Collectively, these results indicate that XRF-1021 is effective in PO-induced acute HUA rats, with a minimal effective dose of 4.9 mg/kg and a delayed onset relative to FEB, consistent with a uricosuric mechanism. In terms of onset, durability, and extent of serum UA reduction, XRF-1021 showed efficacy comparable to 10 mg/kg BEM.

### 2.7. XRF-1021 Lowers UA Levels in a Chronic HUA Quail Model

**Figure 10:**
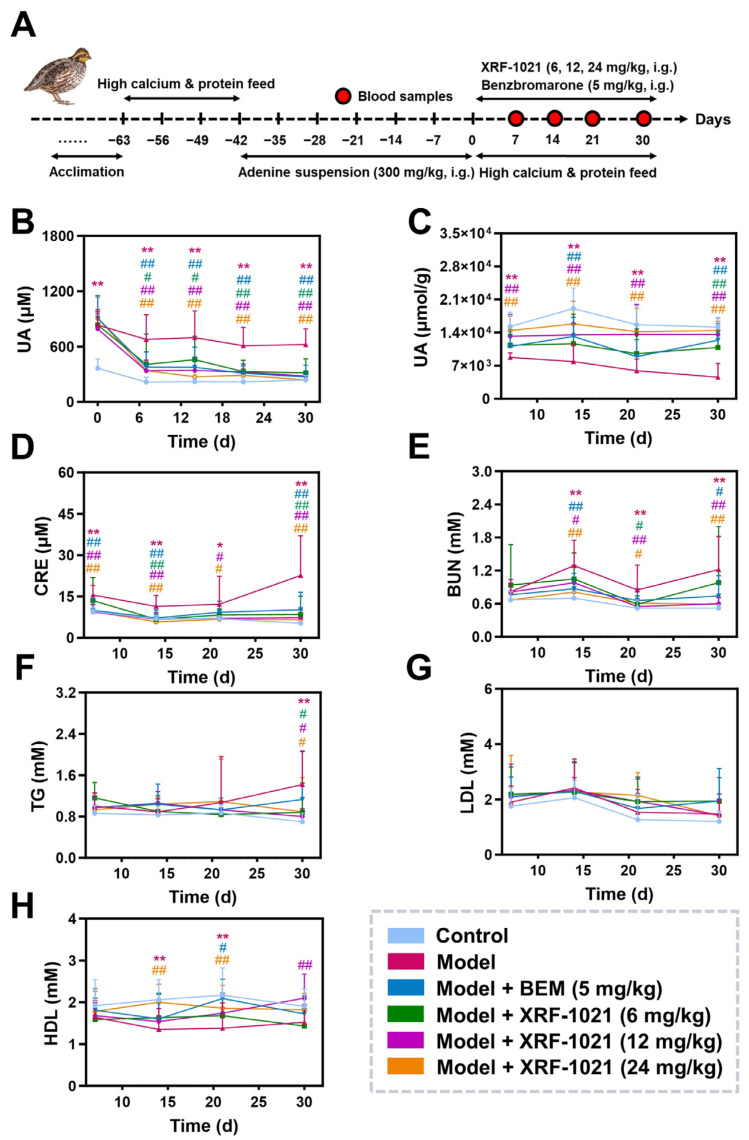
(**A**) Schematic of treatment regimen for chronic HUA quail model. The time-dependent UA content in (**B**) serum and (**C**) feces-urine from treated quails (*n* = 10). Kinetic curves of blood biochemical indicators level (**D**) CRE, (**E**) BUN, (**F**) TG, (**G**) LDL, and (**H**) HDL in serum from treated quails (*n* = 10). Data are presented as mean ± SD. For data that satisfied normality and homogeneity of variance, the one-way ANOVA followed by Dunnett’s *t*-test was applied. For data that did not meet the normality assumption, the Kruskal–Wallis followed by Dunn’s post hoc test was used. (* *p* < 0.05, ** *p* < 0.01, Model vs. Control; ^#^ *p* < 0.05, ^##^ *p* < 0.01, vs. Model).

HUA is typically a chronic, progressive disorder driven by metabolic abnormalities, including dysregulated purine and lipid metabolism. To evaluate the in vivo therapeutic efficacy of XRF-1021 in chronic HUA, we established a quail model induced by a high-calcium/high-protein diet combined with oral adenine suspension. Quails were treated with BEM or XRF-1021, and serum UA, cumulative feces-urine UA excretion, renal function indices, and lipid metabolism parameters were assessed (Figure 10A).

Consistent with its uricosuric profile, XRF-1021 produced a sustained reduction in serum UA that was broadly comparable to BEM in terms of onset, duration, and magnitude. After 7 days of dosing, 6, 12, and 24 mg/kg XRF-1021 and 5 mg/kg BEM significantly decreased serum UA by 39.8%, 49.8%, 50.1%, and 44.4%, respectively, with XRF-1021 showing a clear dose–response relationship. These significant UA-lowering effects persisted to day 30, with serum UA reductions of 49.4%, 55.0%, 61.6%, and 56.6%, respectively (Figure 10B). In parallel, XRF-1021 increased cumulative UA excretion to an extent comparable with BEM. On day 30, total UA excretion in the 6, 12, and 24 mg/kg XRF-1021 groups and the 5 mg/kg BEM group increased by 1.4-, 2.0-, 2.1-, and 1.7-fold versus the model group, respectively, again demonstrating dose dependence for XRF-1021 (Figure 10C). Notably, although XRF-1021 inhibited URAT1 less potently than BEM, its comparable inhibition of GLUT9 may provide a cooperative effect, resulting in similar in vivo UA-lowering efficacy.

Both BEM and XRF-1021 ameliorated renal dysfunction in chronic HUA quails, with XRF-1021 showing a modestly greater renoprotective effect. In the model group, serum creatinine (CRE) and blood urea nitrogen (BUN) were significantly elevated by 3.3- and 1.3-fold relative to normal controls. After 30 days of treatment, CRE decreased by 62.4%, 67.7%, and 70.8% in the 6, 12, and 24 mg/kg XRF-1021 groups, respectively, and by 54.9% in the BEM group (Figure 10D), corresponding BUN reductions were 19.7%, 50.8%, 51.6%, and 39.3% (Figure 10E). In contrast, XRF-1021 did not significantly modulate triglycerides (TG), low-density lipoprotein (LDL), or high-density lipoprotein (HDL) (Figure 10F–H), which may reflect its primary UA-lowering activity without direct intervention in upstream metabolic risk factors.

Overall, at equivalent doses, XRF-1021 exhibited slightly weaker UA-lowering efficacy than BEM in chronic HUA quails but provided somewhat stronger improvement in renal functional injury. This profile is consistent with additional kidney-protective benefits potentially mediated by HIPK2 inhibition.

### 2.8. Therapeutic Efficacy of XRF-1021 in an Adenine-Induced Mouse Model of Chronic Renal Fibrosis Complicated with HUA

**Figure 11:**
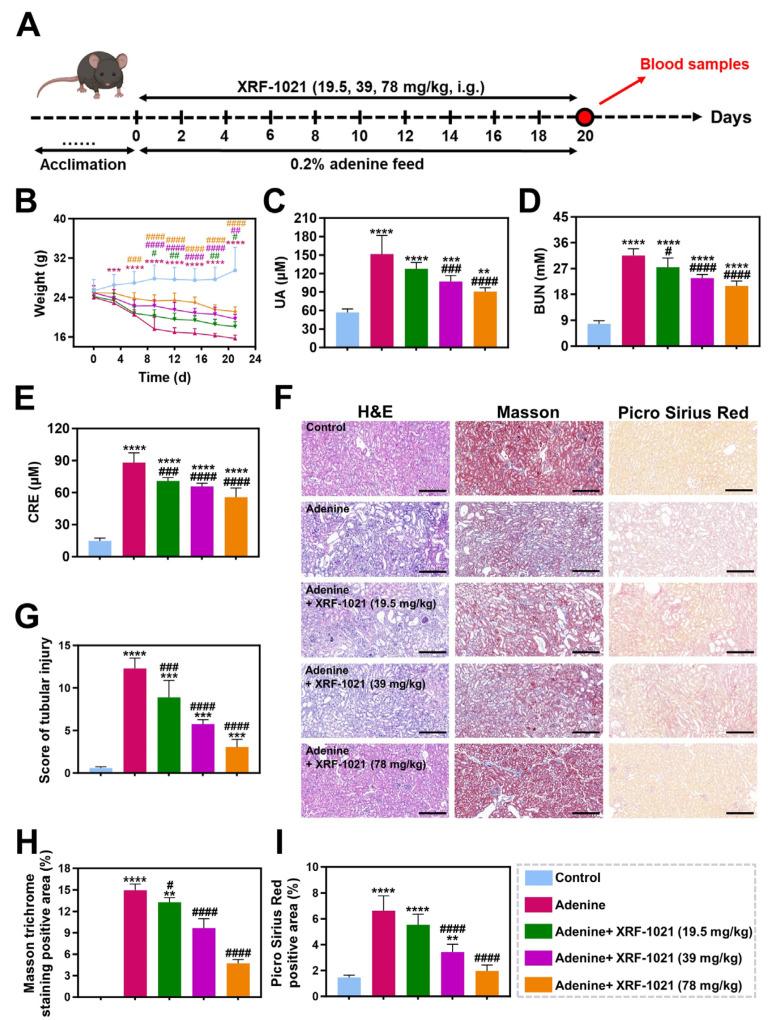
(**A**) Schematic of treatment regimen for adenine-induced HUA mouse model. (**B**) Average body weights of mice with different treatments. (*n* = 8). The level of biochemical indexes, including (**C**) UA, (**D**) BUN, and (**E**) CRE in serum from treated mice (*n* = 5). (**F**) Representative H&E-, Masson-, and Picro Sirius Red-stained histopathological images of kidney from mice in different treatment groups. Scale bar = 200 μm. (**G**) The score of pathological damage renal tubules in H&E staining (*n* = 5). (**H**) The area score of renal fibrosis in Masson staining and (**I**) Picro Sirius Red staining (*n* = 5). Data are presented as mean ± SD. Normality and homogeneity of variances were satisfied. Statistical significance was determined by one-way ANOVA followed by Dunnett’s *t*-test (** *p* < 0.01, *** *p* < 0.001, **** *p* < 0.0001, vs. Control; ^#^ *p* < 0.05, ^##^ *p* < 0.01, ^###^ *p* < 0.001, and ^####^ *p* < 0.0001, vs. Adenine).

TIF contributes to HUA by impairing tubular function, disrupting the expression of UA transporters, and increasing endogenous UA synthesis [41]. Our previous studies demonstrated that XRF-1021 possesses potent anti-TIF activity and significantly improves renal function [42]. To further explore its therapeutic potential, we employed XRF-1021 in a mouse chronic renal fibrosis model induced by 0.2% adenine-containing diet, aiming to evaluate its efficacy against fibrosis-associated HUA (Figure 11A). During treatment, body weight was monitored at regular intervals (Figure 11B). Mice in the model group exhibited pronounced weight loss, likely due to protein depletion and fat consumption caused by fibrotic progression. XRF-1021 conferred a dose-dependent protective effect on body weight, suggesting that it may effectively delay the deterioration in health caused by TIF.

To further assess therapeutic outcomes, blood samples were collected at the end of treatment for UA and biochemical analysis (Figure 11C–E). The adenine group displayed a ~15-fold increase in serum UA, along with marked elevations in serum CRE and BUN compared with controls, caused by severe renal impairment and associated metabolic disturbance. However, XRF-1021 not only dose-dependently reduced serum UA levels in mice, but also improved biochemical indices of hepatic and renal function, indicating that it exerts a uricosuric effect while concurrently conferring hepatorenal protection.

Notably, beyond promoting UA excretion through inhibition of URAT1 and GLUT9, XRF-1021 is a potent HIPK2 inhibitor with strong anti-fibrotic activity. To evaluate its impact on renal fibrogenesis, kidney sections from each group were subjected to H&E, Masson’s trichrome, and PSR staining (Figure 11F). H&E staining revealed that prolonged administration of 0.2% adenine induced prominent tubular dilatation, loss of the brush border, and marked interstitial inflammatory infiltration, whereas oral XRF-1021 substantially improved renal architecture and reduced inflammatory cell infiltration. Consistently, tubular injury scoring showed that increasing the dose of XRF-1021 from 19.5 to 78 mg/kg decreased pathology scores by 27.7–75.2% relative to the adenine group (Figure 11G). In parallel, Masson’s trichrome (collagen stained blue) and PSR staining (collagen fibers visualized under polarized light) both demonstrated a dose-dependent reduction in collagen-positive areas following XRF-1021 treatment, supporting robust suppression of renal fibrosis progression (Figure 11H–I).

**Figure 12:**
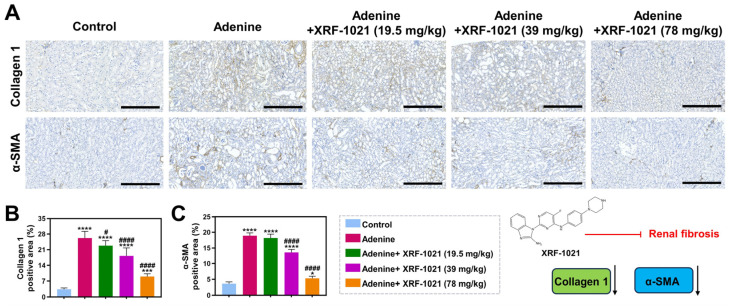
Representative immunohistochemical images showing (**A**) Collagen 1 and α-SMA expression in mouse kidney sections across different treatment groups. Scale bar = 200 μm. Quantification of (**B**) Collagen 1 and (**C**) α-SMA staining is presented (*n* = 5). Data are presented as mean ± SD. Normality and homogeneity of variances were satisfied. Statistical significance was determined by one-way ANOVA followed by Dunnett’s *t*-test (* *p* < 0.05, *** *p* < 0.001, **** *p* < 0.0001, vs. Control; ^#^ *p* < 0.05, and ^####^ *p* < 0.0001, vs. Adenine).

Furthermore, immunohistochemistry showed that XRF-1021 markedly downregulated the renal expression of Collagen I and α-SMA (Figure 12A–C), two canonical markers of extracellular matrix deposition and myofibroblast activation, respectively.

Collectively, these findings demonstrate that XRF-1021 possesses dual pharmacological activities for HUA management: it enhances UA excretion while mitigating renal fibrogenesis to preserve kidney function, thereby sustaining its UA-lowering efficacy, and highlights its translational potential for the treatment of secondary HUA associated with renal fibrosis.

### 2.9. In Vivo Acute and Long-Term Toxicity Evaluation of XRF-1021

As HUA is a chronic metabolic disorder that typically requires long-term pharmacotherapy to maintain UA homeostasis, we evaluated the safety of XRF-1021 to support its translational potential.

Acute toxicity was first assessed in SD rats following a single oral administration of XRF-1021 at doses corresponding to 48–271-fold the therapeutic range. Survival curves for male and female rats are shown in Figure S3A,B, indicated the maximum tolerated (MTD) dose was 773 mg/kg, and the LD_50_ values were 2345.4 mg/kg in males and 1078.9 mg/kg in females. This sex difference may be attributable to preferential accumulation of XRF-1021 in the uterus and ovaries, potentially perturbing hormonal homeostasis. Body weight, a sensitive indicator of overall health status, also showed a dose-related decrease following XRF-1021 administration (Figure S3C,D).

Repeated dosing was used to mimic the clinical requirement for long-term treatment and to assess cumulative and subchronic toxicity. SD rats received XRF-1021 by oral gavage at 144, 289, or 433 mg/kg once daily for 4 weeks, corresponding to approximately 9-, 18-, and 27-fold the efficacious dose, followed by a 4-week recovery period. No obvious changes in body weight were observed in any treatment group during dosing (Figure S4A,B). Food consumption differed from control group in females across the dosing period, while males showed transient decreases at certain time points (Figure S4C,D).

Organ coefficients (organ weight/body weight) indicated dose-related, organ-specific effects at the end of dosing: the spleen coefficient was reduced in males at 289 mg/kg, whereas kidney and liver coefficients were increased at 433 mg/kg, an elevated kidney coefficient was also observed in females at the mid dose (Figure S4E–J). These alterations were largely reversible during the withdrawal phase, with substantial recovery evident within 2 weeks, suggesting that XRF-1021 may induce mild, transient organ swelling/inflammation at higher exposures but overall exhibits a favorable long-term safety profile.

## 3. Discussion

The prevalence of HUA continues to rise globally, with an increasing incidence among younger populations, making it a significant public health concern [43]. HUA is frequently associated with chronic conditions such as obesity, chronic kidney disease (CKD), diabetes, and hypertension, underscoring the need for integrated therapeutic strategies [44]. Persistent HUA induces progressive renal dysfunction through multiple pathways, impairing UA metabolism [45]. Clinical evidence further indicates that HUA is a major driver of TIF [46], while HIPK2 has emerged as a critical antifibrotic target. Building upon our previous work on XRF-1021, we investigated its mechanism of UA reduction and renal protection.

XRF-1021 is not a uricase activator, indicating that the UA-lowering effect observed in mice is not attributable to activation of endogenous uricase. Moreover, the cardiotoxicity of XOIs has been attributed to inhibition of XOD in purine catabolism, which can lead to accumulation of upstream precursors (e.g., hypoxanthine), disruption of cardiomyocyte nitroso–redox homeostasis, ultimately causing cellular injury and electrophysiological abnormalities [47,48]. XRF-1021 does not inhibit UA biosynthesis, thereby avoiding hypoxanthine accumulation and the associated oxidative stress-mediated cardiovascular injury.

Notably, XRF-1021 inhibits URAT1 with potency comparable to LES, although it is substantially less potent than BEM. However, in vivo pharmacology in a PO-induced acute HUA rat model indicated that XRF-1021 achieved UA-lowering efficacy comparable to BEM. This apparent discrepancy may be explained by the additional inhibitory activity of XRF-1021 against GLUT9, which is supported by the shRNA-mediated URAT1 or GLUT9 knockdown rat model. Given that URAT1 and GLUT9 displayed comparable regulatory effects on serum UA in vivo, these data further support that XRF-1021 simultaneously inhibits both transporters and thereby exerts a synergistic UA-lowering effect. Notably, dual-transporter inhibition may further enhance UA reduction, but clinical research data is required for validation.

Furthermore, the molecular docking revealed that XRF-1021 firmly binds to hURAT1 and hGLUT9 by establishing more extensive hydrophobic interactions, particularly π–π and π–alkyl contacts, attributable to its rigid, highly conjugated polyheteroaromatic scaffold. These predicted binding modes not only provide a structural rationale for target engagement but also lay a foundation for future structure–activity relationship optimization of XRF-1021.

Due to the high structural and physicochemical similarity among UA transporters, small-molecule inhibitors often display non-specific binding to multiple transporters, resulting in uncertain efficacy and increased safety risks. This remains a major challenge in the development of UA transporter inhibitors. In contrast, our study demonstrated that XRF-1021 exhibited no significant inhibitory effects on OAT3, OAT4, or the intestinal UA transporter ABCG2 in functional assays, indicating minimal off-target activity and improved target selectivity. Moreover, XRF-1021 upregulated the expression of OAT3, OAT4 and NPT4 in renal tubules, thereby enhancing UA secretion pathways and potentially facilitating the excretion of concomitant drugs and endogenous toxins. Although the novel URAT1 inhibitor dotinurad has been shown to exhibit higher target selectivity with minimal inhibition of OAT1, OAT3, and ABCG2 [49], our findings provide additional insight into strategies for improving the selectivity of both URAT1 and GLUT9 inhibitors.

In SD rats, XRF-1021 exhibited dose-proportional systemic exposure and preferential renal distribution, maintaining relatively high kidney concentrations for up to 24 h post-dose, supporting renal targeting without additional formulation complexity. However, at 24 h post-dose, appreciable accumulation was also observed in the uterus and ovary. This may be attributable to dissociation of XRF-1021 in systemic circulation (pH 7.4), releasing a more lipophilic free-base form that preferentially partitions into lipid-rich tissues, consistent with the increased exposure observed in adipose tissue. These findings highlight the need to closely monitor potential reproductive toxicity in subsequent nonclinical studies, which will also inform molecule optimization and clinical dose selection.

In a PO-induced acute HUA rat model, XRF-1021 achieved an onset, duration, and overall UA-lowering efficacy comparable to BEM, despite requiring only one-fifth of the FEB-equivalent UA-lowering dose. This is consistent with a cooperative mechanism involving inhibition of URAT1 and GLUT9 (GLUT9a/GLUT9b) together with enhanced UA secretion via upregulation of NPT4 and OAT3. Because HUA frequently co-occurs with CKD and TIF is a key driver of renal dysfunction [50], we further evaluated XRF-1021 in chronic models. In hyperuricemic quail, XRF-1021 lowered serum UA slightly less than BEM but produced greater improvements in renal injury indices. In an adenine-induced, fibrosis-associated HUA mouse model, XRF-1021 attenuated TIF by reducing renal collagen deposition and improved renal function. Notably, our prior unilateral ureteral obstruction (UUO) rat study—an HUA-independent fibrosis model—demonstrated that XRF-1021 directly suppresses renal fibrogenesis, as evidenced by decreased renal HIPK2, α-SMA, and collagen I [26]. Together, these findings suggest that XRF-1021 may mitigate renal fibrosis through both indirect UA lowering and direct inhibition of the HIPK2-driven fibrotic signaling cascade. These results also warrant further investigation into whether HIPK2 modulation contributes additional anti-inflammatory benefits in acute gouty inflammation [51].

In addition, acute toxicity studies indicated a relatively wide therapeutic window (4.9–733 mg/kg). Across repeated dosing at three dose levels (144, 289, and 433 mg/kg) and during the recovery period, no evident changes were observed in body weight or in the organ coefficients of the spleen, liver, and kidney, suggesting a favorable safety margin and potentially acceptable safety under chronic dosing. Nevertheless, toxicokinetic investigations and systematic evaluation of reproductive and genotoxicity are still warranted to further characterize the toxicological profile of XRF-1021.

Taken together, this study provides the first demonstration of the therapeutic potential of a HIPK2-structure–based small-molecule inhibitor in HUA. XRF-1021 binds tightly to the UA-binding sites of URAT1 and GLUT9, prevents conformational transitions, and thereby inhibits transporter activity to promote UA excretion. Importantly, XRF-1021 showed no interference with other transporters, ensuring normal drug and endogenous metabolite clearance and enhancing overall biosafety. XRF-1021 exhibited dose-dependent systematical exposure with pronounced renal targeting. Furthermore, through its intrinsic UA lowering and anti-TIF activity, XRF-1021 protects renal function and overcomes the inherent limitations of conventional uricosurics. Meanwhile, XRF-1021 maintained a favorable safety profile during long-term dosing. These findings highlight XRF-1021 as a novel paradigm for designing next-generation targeted therapeutics for HUA.

## 4. Materials and Methods

### 4.1. Experiments Reagents

XRF-1021 (molecular formula: C_21_H_21_FN_8_·2HCl, MW: 477.37, pK_a_ = 4.9 ± 0.9), an off-white powder synthesized in our laboratory, was dissolved in methanol for high-performance liquid chromatography (HPLC) analysis. The chromatographic conditions are detailed in Table 2. A YMC-Pack Pro C18 column (150 × 4.6 mm, 3 μm) was used with a 10 μL injection volume, a flow rate of 0.8 mL/min, and a column temperature of 35 °C. Detection was carried out using a UV detector at 254 nm, with the sample eluting at 15.636 min. The HPLC analysis revealed a purity of 98–102% on an anhydrous basis. BEM and LES were obtained from Shanghai Bidepharm Co., Ltd. (Shanghai, China). [8-^14^C]-UA was purchased from American Radiolabeled Chemicals (Saint Louis, MO, USA). The UA, 6-carboxyfluorescein (6-CFL) and potassium oxonate (PO) were acquired from Shanghai Aladdin Bio-Chem Technology Co., Ltd. (Shanghai, China). The Dulbecco’s Modified Eagle Medium (DMEM) and phosphate-buffered saline (PBS) were purchased from ProCell (Wuhan, China). The fetal bovine serum (FBS) was purchased from ExCell Bio Group (Shanghai, China). The 0.2% adenine feed was provided by TROPHIC Animal Feed High-tech Co., Ltd. (Nantong, China). The Lipofectamine 3000 was purchased from Invitrogen (Carlsbad, CA, USA). The Lipofectamine TM 2000 and poly-D-lysine were obtained from Sigma-Aldrich (Saint Louis, MO, USA). The uricase activity assay kit was supplied by Beijing Solarbio Science & Technology Co., Ltd. (Beijing, China), and xanthine oxidase activity assay kit was purchase from Boxbio (Beijing, China). The antibodies used in Western Blot were listed in Table 3. The shRNA-expressing adenovirus was constructed by Jiangxi Zvast-Biotechnology Co., Ltd. (Nanchang, China).

### 4.2. Cells and Transfection

HEK293T and HK-2 cell lines were obtained from National Collection of Authenticated Cell Cultures (Shanghai, China), cultured, and grown in DMEM. All the mediums were supplied with 10% FBS and 1% streptomycin/penicillin, and cells were incubated in a humidified atmosphere with 5% CO_2_ at 37 °C.

HEK293T cells were seeded in 96-well plates and incubated overnight. At 70–90% confluence, HEK293T cells were cotransfected with plasmids (pcDNA3.1) expressing URAT1, GLUT9a, GLUT9b, OAT1, OAT3, OAT4, and ABCG2 along with EGFP by using Lipofectamine 3000 or Lipofectamine TM 2000. Then, after 24-h-transfection, the fluorescent tag EGFP was observed as a marker to ensure that the plasmid was successfully transfected into the cells. Successfully transfected cells expressing URAT1 and ABCG2 were used to perform [8-^14^C] UA uptake assays, while transport efficacy of the cells expressing OAT1, OAT3 and OAT4 were measured by 6-CFL. Additionally, the cells expressing GLUT9a and GLUT9b were split using trypsin and replated onto 10 mm coverslips coated with 0.1 mg/mL poly-D-lysine for electrophysiology experiments.

### 4.3. Animals

The C57BL/6J mice, Sprague-Dawley rats and Duffek quail were purchased from Hunan SJA Laboratory Animal CO., Ltd. (Hunan, China) and Nanjing Tianpeng Biotechnology Co., Ltd. (Nanjing, China), housed under specific pathogen-free conditions.

### 4.4. Uricase Inhibition Assay

A uricase working solution (0.02 mg/mL) was prepared. PO or XRF-1021 was added to the enzyme solution to yield final concentrations of 3, 10, 30, 50, 100, and 300 μM. Uricase activity was immediately determined using a uricase activity assay kit according to the manufacturer’s instructions. Absorbance was measured at 505 nm with a microplate reader. Enzyme activity was calculated from the absorbance readings, and the inhibition rate of the test compound was calculated using the following equation:

$$Uricase\;inhibition\;rate\;(\%)=\frac{{Activity}_{blank}-{Activity}_{test}}{{Activity}_{blank}}\times 100\%$$

### 4.5. XOD Inhibition Assay

The XOD working solution (0.875 mg/mL) was prepared. Allopurinol or XRF-1021 was added to the enzyme solution to yield final concentrations of 0, 0.1, 0.3, 1, 3, and 10 μM. The XOD activity was immediately measured using a xanthine oxidase activity assay kit according to the manufacturer’s instructions. Absorbance was recorded at 290 nm using a microplate reader. XOD activity was calculated from the absorbance readings, and the inhibition rate of the test compound was determined using the following equation:

$$XOD\;inhibition\;rate\;(\%)=\frac{{Activity}_{blank}-{Activity}_{test}}{{Activity}_{blank}}\times 100\%$$

### 4.6. [8-^14^C] UA Uptake Assays of URAT1

URAT1-HEK293T cells were incubated with varying concentrations of BEM, LES, or XRF-1021 for 30 min. Subsequently, cells were incubated for 10 min with UA uptake buffer containing 50 μM [8-^14^C]-UA, 125 mM sodium gluconate, 4.8 mM potassium gluconate, 1.4 mM calcium gluconate, 1.2 mM monobasic potassium phosphate, 1.2 mM magnesium sulfate, 5.6 mM glucose, and 25 mM HEPES. The reaction was terminated by washing the cells three times with ice-cold DPBS. Cells were then lysed by adding 100 μL of 0.1 M NaOH. Intracellular radioactivity was measured using a liquid scintillation counter (MicroBeta 2450, PerkinElmer, Waltham, MA, USA) after adding 200 μL of scintillation fluid. The inhibition rate was calculated according to the following formula:

$$Inhibition\;rate\left(\%\right)=\frac{{CPM}_{control}-{CPM}_{test}}{{CPM}_{control}-{CPM}_{blank}}\times 100\%$$

### 4.7. Electrophysiological Recordings of GLUT9a and GLUT9b Currents

Currents were recorded 18 h after transfection by the whole-cell patch-clamp technique and measured with a MultiClamp 700B patch-clamp amplifier, Digidata 1550B digitizer, and pClamp 10 software (Molecular Devices, Sunnyvale, Santa Clara, CA, USA). The electrodes were pulled from borosilicate glass capillaries (BF150-110-10, Sutter Instruments, Novato, CA, USA) and had a resistance of 2 to 5 MΩ when filled with intracellular solution. The intracellular solution contained 140 mM KCl, 1 mM MgCl_2_, 5 mM EGTA, and 10 mM HEPES (pH adjusted to 7.4 with KOH), whereas the extracellular solution was composed of 140 mM NaCl, 5 mM KCl, 1 mM MgCl_2_, 2 mM CaCl_2_, 10 mM HEPES, and 10 mM D-glucose (pH adjusted to 7.4 with NaOH). The stock solutions were diluted with extracellular solution containing 1 mM UA extracellular solution to obtain the final concentrations before the experiments. During the experiments, a constant perfusion of solution (control or solution containing XRF-1021 at varying concentration) was delivered by a homemade perfusion device, allowing the rapid solution exchange with an approximate flow velocity. The remaining activity was calculated according to the following formula:

$$Activity\left(\%\right)=\frac{I}{I_{0}}\times 100\%$$

### 4.8. URAT1/GLUT9 Knockdown in Rats by shRNA-Expressing Adenovirus

To investigate the UA-lowering mechanism of XRF-1021, a HUA model was established in SD rats via intraperitoneal injection of PO (300 mg/kg) suspension, combined with targeted gene interference. Three weeks prior to PO administration, rats received bilateral renal pelvis injections of 50 μL adenovirus carrying shRNA targeting URAT1, GLUT9, or negative control (NC). All rats were randomly divided into eight groups (*n* = 4 per group) and treated as follows: (1) Control; (2) PO (300 mg/kg, i.p.); (3) PO (300 mg/kg, i.p.) + XRF-1021 (16 mg/kg, i.p.); (4) NC + PO (300 mg/kg, i.p.); (5) URAT1 sh1 + PO (300 mg/kg, i.p.); (6) URAT1 sh1 + PO (300 mg/kg, i.p.) + XRF-1021 (16 mg/kg, i.g.); (7) GLUT9 sh1 + PO (300 mg/kg, i.p.); and (8) GLUT9 sh1 + PO (300 mg/kg, i.p.) + XRF-1021 (16 mg/kg, i.g.). Blood samples were collected at 1 day before surgery and at 2-, 4-, 6-, and 8-h post-treatment. Serum UAlevels were measured using a 7600 automated biochemistry analyzer (HITACHI, Tokyo, Japan). The shRNA sequences were listed in Table 4.

### 4.9. Molecular Docking and Molecular Dynamics Simulations

URAT1 (PDB ID: 9JDY) was downloaded from RCSB PDB and preprocessed in PyMOL 3.0 (Schrödinger, LLC): ligands were removed, missing residues rebuilt, and protonation adjusted. GLUT9a/b sequences (UniProt Q9NRM0) were modeled by AlphaFold 3 (DeepMind) and energy-minimized. For molecular docking, the grid box was centered at the geometric center of the substrate-binding site of each protein. The grid-center coordinates and grid size for URAT1 and GLUT9a/b are provided in Table 5. The XRF-1021 ligand was minimized in Avogadro (Avogadro Project) and converted to PDBQT. Receptors and ligand were prepared in AutoDockTools 4 (The Scripps Research Institute) by adding polar hydrogens, assigning Gasteiger charges, and defining rotatable bonds. Docking was performed with AutoDock Vina 1.2.5 (The Scripps Research Institute) using a global grid (0.375 Å spacing, exhaustiveness 8), the top-scoring pose was selected. Interactions were analyzed with Discovery Studio Visualizer 2024 (Dassault Systèmes BIOVIA) and final figures rendered in PyMOL 3.0 (Schrödinger, LLC).

MD simulations were performed using the GROMACS software package (v2025.1). The systems were solvated in a periodic truncated octahedral box with TIP3P water molecules, ensuring a minimum buffer distance of 10 Å between the solute and the box edges. Counterions were added as needed to neutralize the overall charge. The AMBER ff99SB-ILDN force field was employed for protein. Bonds involving hydrogen atoms were constrained using the LINCS algorithm with a fourth-order expansion and one iteration. Non-bonded interactions were treated with a twin-range cutoff scheme: van der Waals and short-range electrostatic interactions were truncated at 10 Å, while long-range electrostatics were handled using the Particle Mesh Ewald (PME) method with a grid spacing of 1.2 Å and fourth-order spline interpolation. The systems underwent energy minimization via the steepest descent algorithm for up to 50,000 steps or until convergence (force tolerance of 1000 kJ mol^−1^ nm^−1^), followed by conjugate gradient minimization if necessary. Subsequently, the systems were equilibrated in the NVT ensemble for 2500 ps at 300 K using the velocity-rescaling thermostat (τ_T = 0.1 ps), with positional restraints on heavy atoms (1000 kJ mol^−1^ nm^−2^). This was followed by 5000 ps of NPT equilibration at 1 bar using the Parrinello-Rahman barostat (τ_P = 2.0 ps), maintaining the same restraints. Production simulations were then conducted in the NPT ensemble for 50 ns with an integration time step of 2 fs, employing the leap-frog integrator. Coordinates were saved every 2 ps for analysis.

### 4.10. 6-CFL Uptake Assays of OAT3 and OAT4

OAT3-HEK293T and OAT4-HEK293T cells were incubated separately with 90 μL HBSS (model), 10 μM XRF-1021, or LES for 30 min at 37 °C. Subsequently, 10 μL of 1 mM 6-carboxyfluorescein (6-CFL) was added into each well, and the plates were incubated for an additional 20 min in the dark. After incubation, the reaction mixture was removed, and cells were washed twice with ice-cold DPBS to terminate the reaction. Cell lysis was performed by adding 150 μL of 100 μM NaOH solution to each well, followed by incubation for 15 min at room temperature. Finally, fluorescence intensity was measured using a multifunctional microplate reader. The inhibition rates for each compound were calculated using the following formula:

$$Inhibition\;rate\left(\%\right)=(1-\frac{F_{test}-F_{0}}{F_{model}-F_{0}})\times 100\%$$

### 4.11. [8-^14^C] UA Uptake Assays of ABCG2

ABCG2-HEK293T membrane vesicles were prepared as follows: harvested cells were resuspended in TS buffer (10 mM Tris-HEPES, 250 mM sucrose, pH = 7.4), and the suspension was transferred into a Z-shaped centrifugation tube. Samples were briefly centrifuged at 16,000× *g* for 30 s, and the flow-through from the column was discarded. The retained cells were vortexed to resuspend, then centrifuged at 700× *g* for 1 min to remove nuclei. The resulting supernatant was centrifuged at 16,000× *g* for 30 min at 4 °C to separate the cytoplasmic fraction. The pellet was resuspended in TS buffer and subsequently centrifuged at 100,000× *g* for 1 h at 4 °C. Finally, the membrane pellet was collected and resuspended in TS buffer and stored until further use.

Fifty microliters of membrane vesicle mixture containing 20 μM [8-^14^C]-UA was incubated with XRF-1021 or LES for 30 min at 37 °C, and the reaction was started by adding ATP (with a final concentration of 4 mM). Fifteen minutes later, the reaction was stopped by adding 200 μL of ice-cold DPBS buffer, and this solution was added to a 0.45 μm Ultrafree filter (Merck Millipore, Darmstadt, Germany). The filter was immediately centrifuged at 3000× *g* for 20 s. Following two washing steps with ice-cold Hanks’ balanced salt solution, the vesicles were solubilized by the addition of 200 μL of 0.1 M NaOH. Radioactivity was determined with a liquid scintillation counter (PerkinElmer, Boston, MA, USA) after the addition of 0.5 mL of scintillant, and the inhibition rate was calculated according to the following formula:

$$Inhibition\;rate\left(\%\right)=(1-\frac{{CPM}_{test}-{CPM}_{blank}}{{CPM}_{model}-{CPM}_{blank}})\times 100\%$$

### 4.12. Western Blot

The total cellular proteins from treated HK-2 cells were extracted by RIPA. The protein concentrations were determined by BCA reagent. Equal amounts of protein samples were subjected to SDS-PAGE and transferred to polyvinylidene fluoride membranes. Membranes were probed with the corresponding primary antibodies at 4 °C overnight. The HRP-conjugated goat anti-rabbit secondary antibody was then applied, and the immunoreactive bands were visualized by a ChemiDoc XRS + system (Bio-Rad, Hercules, CA, USA). The quantification of protein expression was quantified by ImageJ software (v2.1.4.7).

### 4.13. Pharmacokinetics and Tissue Distribution in Rats

Eighteen healthy SD rats weighing 200–250 g were used, with equal numbers of males and females. Animals were randomly assigned by body weight into three groups, with six rats per group. XRF-1021 was administered by oral gavage at doses of 7.8 mg/kg, 23.4 mg/kg, and 140.4 mg/kg. Blood samples were collected pre-dose at 0 h and at 15 min, 30 min, 1.0 h, 2.0 h, 3.0 h, 4.0 h, 6.0 h, 8.0 h, 10.0 h, 12.0 h, and 24.0 h post-dose. Plasma was obtained by centrifugation at 4 °C and 1700× *g*, and plasma concentrations of XRF-1021 were quantified by HPLC. Pharmacokinetic parameters were calculated using Phoenix WinNonlin (version 8.0).

For the tissue distribution study, eighteen additional healthy SD rats weighing 200–250 g were used, with equal numbers of males and females. Animals were randomly assigned by body weight into three groups, with six rats per group. Each group received a single oral gavage dose of XRF-1021 at 23.4 mg/kg. Rats were euthanized at 2 h, 6 h or 24 h after dosing, and the heart, liver, spleen, lungs bilaterally, kidneys bilaterally, stomach, small intestine as a 5-cm segment distal to the duodenum, adipose tissue bilaterally, skeletal muscle from the left hind limb, brain, testes bilaterally, ovaries bilaterally, uterus, and serum were collected. Tissues were rinsed three times with pre-cooled 0.9% saline, blotted dry with filter paper, and weighed. Approximately 200 mg of each tissue was transferred to homogenization tubes and homogenized in 50% methanol. The homogenates were centrifuged at 4 °C and 4000 rpm for 10 min, and the resulting supernatants were subjected to HPLC analysis.

### 4.14. In Vivo Therapeutic Efficacy in Murine HUA Model

Acute HUA was induced in male rats by uricase inhibitor PO. To compare XRF-1021 with FEB, the rats were randomly allocated into seven groups (*n* = 8): (1) control, (2) PO (300 mg/kg), (3) FEB (2 mg/kg), and (4–7) XRF-1021 (4.9, 9.8, 20, and 39 mg/kg). To compare XRF-1021 with BEM, the rats were randomly allocated into six groups (*n* = 8): (1) control, (2) PO (300 mg/kg), (3) BEM (10 mg/kg), and (4–6) XRF-1021 (8, 16, and 32 mg/kg). Rats in the model group were intraperitoneally administered 300 mg/kg PO to induce acute HUA. XRF-1021, FEB and BEM were oral administration, respectively. Blood samples were obtained by eye socket at pre-determined time points within 8 h, and then centrifuged at 1700× *g* at 4 °C for 10 min in order to collect the serum. Following this, the level of UA in rats’ serum was measured by a 7600 automated biochemistry analyzer (HITACHI, Tokyo, Japan).

Renal fibrosis was induced in eight-week-old male C57BL/6J mice by feeding a diet containing 0.2% adenine. Mice were randomly divided into three groups as follows (*n* = 8): (1) control, (2) 0.2% adenine, and (3–5) 0.2% adenine + XRF-1021 (19.5, 39, and 78 mg/kg); Body weight was monitored throughout the treatment period. On day 20, blood samples were collected via retro-orbital bleeding. Serum levels of UA and renal function parameters were analyzed using a 7600 automated biochemistry analyzer (HITACHI, Tokyo, Japan). Kidneys were harvested and immersed in 4% formaldehyde, followed by embedding and slicing. Finally, the slides were stained with H&E, Masson, and Picro Sirius Red (PSR) for imaging, respectively. The expression of collagen 1 and α-SMA in kidney tissue was measured by immunochemistry analysis. Using a 200× light microscope, 5 random areas per section were selected for evaluation. In H&E staining, pathology score was performed to evaluate renal tubular and interstitial damage, including tubular dilation, interstitial expansion, tubular atrophy, inflammatory cell infiltration, and protein casts. The tubular injury was scored as follows: 0 score for normal field, 1 score for <25% pathological field, 2 score for 25–50% pathological field, 3 score for 50–75% pathological field, 4 score for >75% pathological field. Masson and PSR staining were utilized to detect and quantify fibrosis, and the extent of collagen deposition was measured and quantified using ImageJ software (v2.1.4.7). The percentage of positive-staining area of immunochemistry images was also analyzed by ImageJ software (v2.1.4.7).

### 4.15. In Vivo Therapeutic Efficacy in Quail HUA Model

A chronic HUA model was established in eight-week-old male Duffek quail by feeding a high-calcium, high-protein diet for 21 days, followed by the oral administration of adenine suspension (300 mg/kg/day) for an additional 42 days. After model establishment, the quail were randomly divided into six groups (*n* = 10): (1) control, (2) adenine, (3) adenine + BEM (5 mg/kg), (4–6) adenine + XRF-1021 (6, 12, and 24 mg/kg). XRF-1021 and BEM were orally administered, respectively. The blood, feces, and urine from treated quails were collected at 7, 14, 21, and 30 days. Blood samples were centrifuged at 1700× *g* for 10 min at 4 °C to obtain serum. The UA levels of serum and feces-urine, renal function parameters, TG, LDL, and HDL were measured using a 7600 automated biochemistry analyzer (HITACHI, Tokyo, Japan) or ELISA kit.

### 4.16. Acute and Long-Term Toxicity of XRF-1021

For the acute toxicity study, fifty healthy SD rats weighing 169.7–215.0 g were used, with equal numbers of males and females. Animals were randomized by body weight into five groups, with ten rats per group. XRF-1021 was administered by oral gavage at single doses of 773 mg/kg, 1190 mg/kg, 1831 mg/kg, 2816 mg/kg, and 4333 mg/kg. Animals were monitored for 14 days after dosing for general clinical signs, and mortality and body weight changes were recorded.

To evaluate long-term toxicity, one hundred and twenty SD rats weighing 180–220 g were used, with equal numbers of males and females. Rats were allocated into four groups, with thirty rats per group: a vehicle control group and three XRF-1021 dose groups at 144 mg/kg, 289 mg/kg, and 433 mg/kg. Animals in the dosing groups received XRF-1021 once daily by oral gavage, seven days per week, for four consecutive weeks. Throughout the dosing period, animals were closely observed for clinical signs, and body weight and food consumption were recorded. At the end of dosing, twenty rats from each group, with equal numbers of males and females, were euthanized immediately. The remaining ten rats in each group were euthanized four weeks after the last dose to assess recovery. The spleen, liver, kidneys, and brain were excised and weighed. Organ coefficients were calculated according to the following equation:

$$Organ\;coefficients\;(\%)=\frac{M_{organ}}{M_{weight}}\times 100\%$$

### 4.17. Statistical Analysis

All statistical analyses were performed using SPSS software version 20.0 (IBM Corp., Armonk, NY, USA). Normality was assessed using the Shapiro–Wilk test, and the homogeneity of variances was assessed using Brown–Forsythe test. If both assumptions were met, parametric tests were applied (ANOVA followed by Dunnett’s *t*-test). If variances were unequal, Welch’s correction was used. If data were not normally distributed, non-parametric tests were applied (Kruskal–Wallis test followed by Dunn’s post hoc test with multiplicity adjustment). A *p*-value of less than 0.05 was considered statistically significant.

## 5. Conclusions

Collectively, mechanistic studies revealed that XRF-1021 is neither an activator of uricase nor an XOI. Instead, XRF-1021 selectively inhibits the two key UA transporters, URAT1 and GLUT9, thereby blocking tubular UA reabsorption and UA efflux into the bloodstream, which consequently leads to a marked reduction in serum UA levels. Meanwhile, XRF-1021 displayed no inhibitory activity against OAT3, OAT4, or ABCG2, while upregulating OAT3, OAT4, and NPT4 expression in renal tubules. This selectivity minimized the risk of drug accumulation and further facilitated UA and endogenous toxin excretion. XRF-1021 showed dose-dependent systemic exposure with preferential renal distribution in SD rats. Across three in vivo models, XRF-1021 rapidly lowered serum UA in acute and chronic HUA and improved outcomes in TIF-associated HUA, while remaining well tolerated during long-term dosing. These findings elucidate the molecular basis by which XRF-1021 promotes UA excretion and confers renal protection, underscore its strong potential for clinical translation, and provide a framework for structure-guided development of HIPK2-targeted small-molecule inhibitors for HUA therapy.

## Figures and Tables

**Table 1:** Pharmacokinetic parameters of XRF-1021 oral administration in SD rats (*n* = 3).

Dosage (mg/kg)	Sex	AUC_0-t_ (h*ng/mL)	C_max_/C_0_ (ng/mL)	t_1/2_ (h)	F (%)	CL/CL_F_ (mL/min/kg)
7.8	F	76.79 ± 47.75	10.91 ± 8.24	5.194 ± 2.431	13.86 ± 8.53	1678 ± 669
	M	36.61 ± 23.38	4.906 ± 3.501	13.27 ± 16.09	6.608 ± 4.221	1963 ± 163
	Total	56.70 ± 39.95	7.547 ± 6.360	9.233 ± 11.200	10.23 ± 7.21	1820 ± 463
23.4	F	171.2 ± 56.2	33.93 ± 24.79	9.910 ± 1.486	10.30 ± 3.38	2088 ± 731
	M	273.6 ± 21.5	67.12 ± 8.41	9.773 ± 3.115	16.46 ± 1.30	1227 ± 103
	Total	222.4 ± 61.9	50.53 ± 22.45	9.841 ± 1.990	13.38 ± 3.72	1658 ± 606
140.4	F	1590 ± 215	200.9 ± 56.8	4.227 ± 0.517	15.94 ± 2.15	1428 ± 175
	M	1581 ± 476	207.6 ± 62.5	5.482 ± 0.885	15.86 ± 4.77	1448 ± 385
	Total	1586 ± 330	204.3 ± 53.5	4.855 ± 0.950	15.90 ± 3.31	1438 ± 237

**Table 2:** Chromatographic conditions of XRF-1021.

Time (min)	KH_2_PO_4_ (17 mM, pH 2.5, 0.15% TFA) (%)	Acetonitrile (%)
0	85	15
30	50	50
40	50	50
41	85	15
50	85	15

**Table 3:** List of antibodies.

Name	Company	Catalog
Anti-OAT3	Proteintech (Wuhan, China)	16844-1-AP
Anti-OAT4	Bioworld Biotech (Dublin, OH, USA)	BS70935
Anti-ABCG2	Proteintech	27286-1-AP
Anti-NPT1	Proteintech	20751-1-AP
Anti-NPT4	Proteintech	MP50837-1
Anti-β-actin	Proteintech	66009-1-Ig

**Table 4:** List of shRNA sequences.

Gene	Top Strand (5′→3′)	Bottom Strand (5′→3′)
URAT1	AATTCGCAAGCCCTAGGAAGCAATATctcgagATATTGCTTCCTAGGGCTTGCTTTTTTG	GATCCAAAAAAGCAAGCCCTAGGAAGCAATATctcgagATATTGCTTCCTAGGGCTTGCG
GLUT9	AATTCGTCTGTAACTGTGTCCATATTCTCGAGAATATGGACACAGTTACAGACTTTTTTG	GATCCAAAAAAGTCTGTAACTGTGTCCATATTCTCGAGAATATGGACACAGTTACAGACG

**Table 5:** The grid-center coordinates and box dimensions for URAT1 and GLUT9a/b.

Protein	Grid Center	Grid Size
URAT1	X: 98.52 Y: 99.25 Z: 87.62	X: 89.48 Y: 50.58 Z: 63.68
GLUT9a	X: −3.76 Y: 7.53 Z: 15.91	X: 80.57 Y: 77.82 Z: 121.25
GLUT9b	X: −9.78 Y: −13.84 Z: 3.77	X: 105.56 Y: 95.79 Z: 75.65

## Data Availability

The original contributions presented in this study are included in the article. Further inquiries can be directed to the corresponding author.

## Supplementary Materials

The following supporting information can be downloaded at: https://www.mdpi.com/article/10.3390/ph19030490/s1, Figure S1. Time evolution of the root-mean-square deviation (RMSD) for the XRF-1021 complexes with (A) URAT1, (B) GLUT9a, and (C) GLUT9b. Per-residue root-mean-square fluctuation (RMSF) of (D) URAT1, (E) GLUT9a, and (F) GLUT9b during the simulations. Time-dependent number of hydrogen bonds formed between XRF-1021 and (G) URAT1, (H) GLUT9a, and (I) GLUT9b; Figure S2. The original Western Blot images of (A) OAT3, (B) OAT4, (C) ABCG2, (D) NPT1, (E) NPT4, and (F) β-actin; Figure S3. Survival curves of (A) male and (B) female rats following a single oral dose of the XRF-1021 at different doses (*n* = 5). Body weight dynamics curves of (C) male and (D) female rats following a single oral dose of the XRF-1021 at different doses (*n* = 2–5); Figure S4. Body weight dynamics curves of (A) male and (B) female rats following a repeated oral dose of the XRF-1021 at different doses (*n* = 15). Time-dependent changes in food consumptions in (C) male and (D) female rats following a repeated oral dose of the XRF-1021 at different doses (*n* = 3). (E) Spleen-, (F) liver-, and (G) kidney organ coefficients in rats after 4 weeks of continuous administration of different doses of XRF-1021 (*n* = 10). (H) Spleen-, (I) liver-, and (J) kidney organ coefficients in rats after 4 weeks of withdrawal from XRF-1021 administration at different doses (*n* = 5).
