# Supplementary material for: A Novel Dual URAT1/GLUT9 Inhibitor Reduces Hyperuricemia by Enhancing Uric Acid Excretion and Attenuating Renal Fibrosis
Source: Pharmaceuticals (Basel). 2026 Mar 16;19(3):490. doi: 10.3390/ph19030490 (PMC13029251; doi:10.3390/ph19030490)
Supplement: Supplementary file 1 [file pharmaceuticals-19-00490-s001.zip › pharmaceuticals-4171041-supplementary.pdf]

A Novel Dual URAT1/GLUT9 Inhibitor Reduces Hyperuricemia by Enhancing Uric Acid Excretion and Attenuating Renal Fibrosis

Weibang Li [1], Ling Li [2,*] and Lidong Zhu [2]

[1] College of Computer Science and Artificial Intelligence, Southwest Minzu University,
Chengdu 610041, China; 21700142@swun.edu.cn

[2] National Key Laboratory of Wireless Communications, University of Electronic Science and Technology of China, Chengdu 611731, China; zld@uestc.edu.cn

* Correspondence: 202111220609@std.uestc.edu.cn

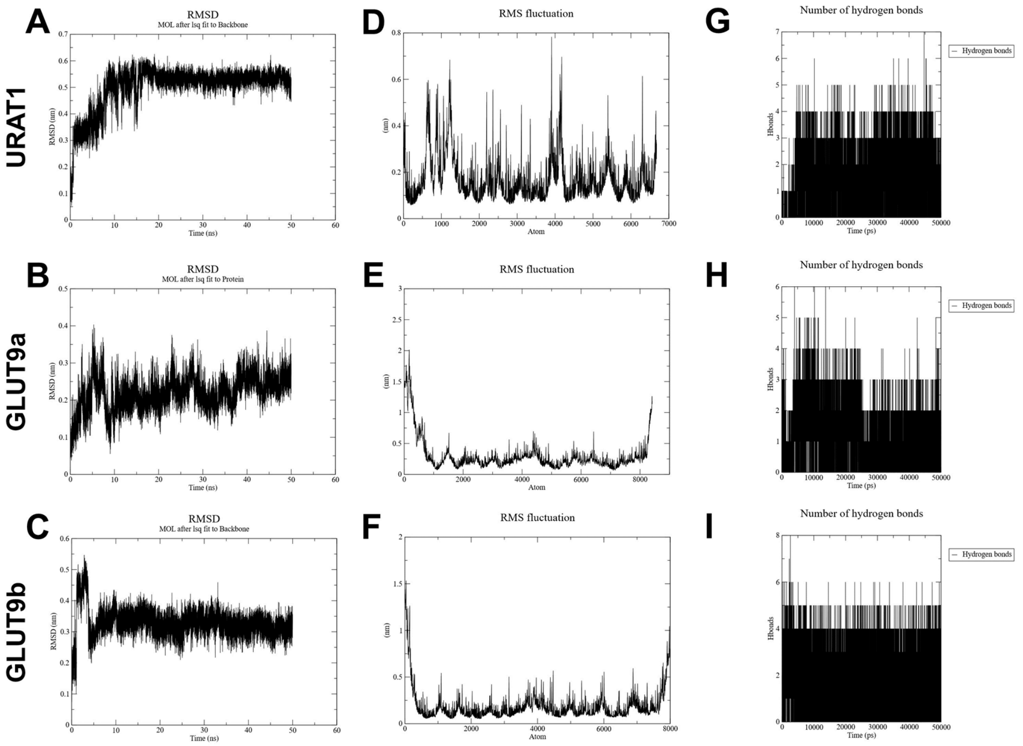

Figure S1. Time evolution of the root-mean-square deviation (RMSD) for the XRF-1021 complexes with (A) URAT1, (B) GLUT9a, and (C) GLUT9b. Per-residue root-mean-square fluctuation (RMSF) of (D) URAT1, (E) GLUT9a, and (F) GLUT9b during the simulations. Time-dependent number of hydrogen bonds formed between XRF-1021 and (G) URAT1, (H) GLUT9a, and (I) GLUT9b.

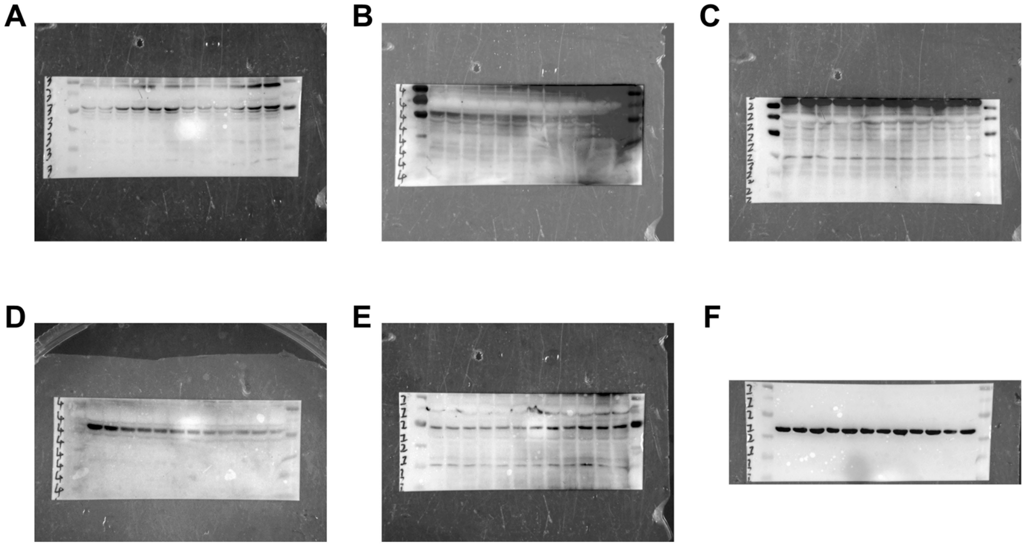

Figure S2. The original Western Blot images of (A) OAT3, (B) OAT4, (C) ABCG2, (D) NPT1, (E) NPT4, and (F) β-actin.

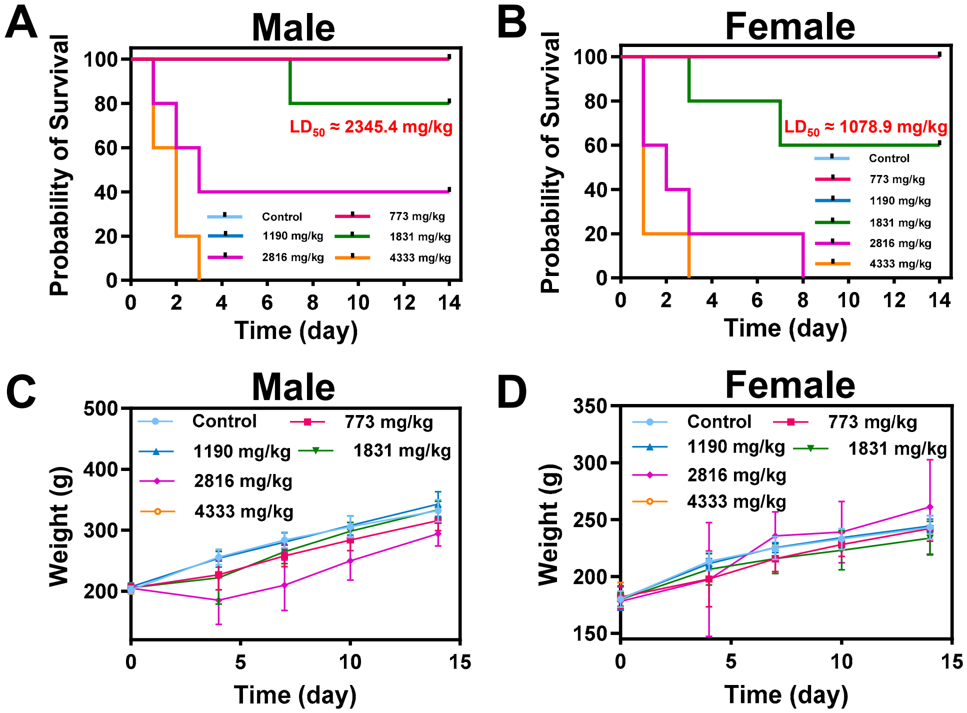

Figure S3. Survival curves of (A) male and (B) female rats following a single oral dose of the XRF-1021 at different doses (n = 5). Body weight dynamics curves of (C) male and (D) female rats following a single oral dose of the XRF-1021 at different

doses (n = 2-5). Data are presented as mean ± SD.

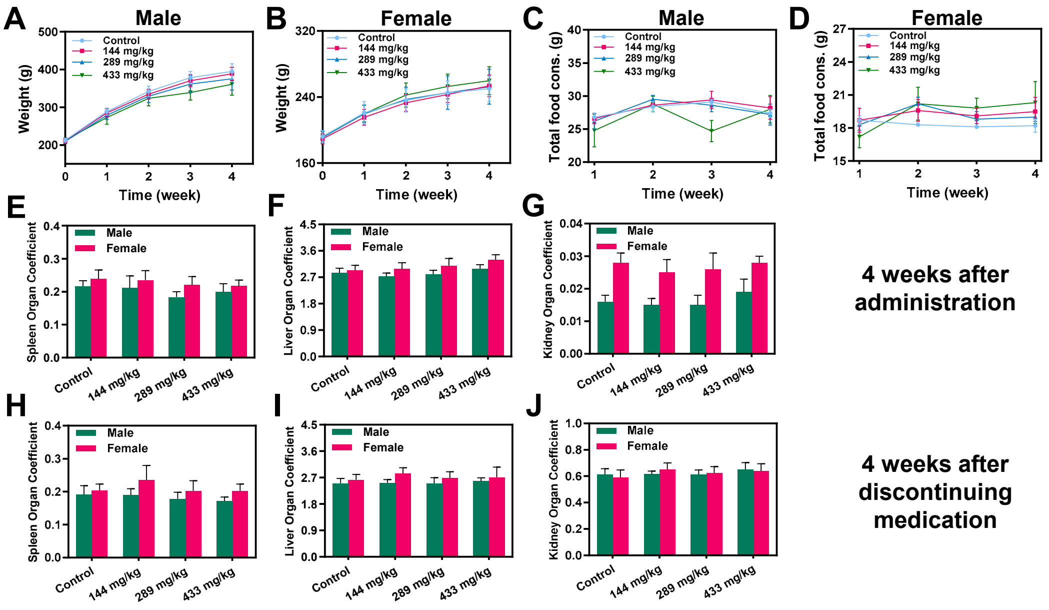

Figure S4. Body weight dynamics curves of (A) male and (B) female rats following a repeated oral dose of the XRF-1021 at different doses (n = 15). Time-dependent changes in food consumptions in (C) male and (D) female rats following a repeated oral dose of the XRF-1021 at different doses (n = 3). (E) Spleen-, (F) liver-, and (G) kidney organ coefficients in rats after 4 weeks of continuous administration of different doses of XRF-1021 (n = 10). (H) Spleen-, (I) liver-, and (J) kidney organ coefficients in rats after 4 weeks of withdrawal from XRF-1021 administration at different doses (n = 5). Data are presented as mean ± SD.
